# Genetic and Antigenic Characterization of Bovine and Porcine Respiratory Coronaviruses Circulating in Western Europe, 2020–2023

**DOI:** 10.3390/v18070705

**Published:** 2026-06-26

**Authors:** Ruth M. Mumo, Anna Parys, Sieglinde Coppens, Nick Vereecke, Sebastiaan Theuns, Bart Pardon, Kristien Van Reeth

**Affiliations:** 1 Laboratory of Virology, Department of Translational Physiology, Infectiology and Public Health, Faculty of Veterinary Medicine, Ghent University, Salisburylaan 133, 9820 Merelbeke, Belgium; ruth3mumo@gmail.com (R.M.M.); 2 National Reference Centre for Respiratory Pathogens, Service of Viral Diseases, Sciensano, Engelandstraat 642, 1050 Brussels, Belgium; 3 PathoSense BV, Poortakkerstraat 41A, 9051 Gent, Belgium; sieglinde.coppens@pathosense.com (S.C.); sebastiaan.theuns@pathosense.com (S.T.); 4 Calf Health Research Group, Clinic for Ruminants, Department of Internal Medicine, Reproduction and Population Medicine, Faculty of Veterinary Medicine, Ghent University, Salisburylaan 133, 9820 Merelbeke, Belgium; bart.pardon@ugent.be

**Keywords:** genetic characterization, coronavirus, antigenic diversity

## Abstract

The 2019 coronavirus disease pandemic (COVID-19) showed how genetic mutations can alter coronavirus characteristics. However, the evolution of livestock coronaviruses remains understudied. We analyzed 15 bovine coronavirus (BCoV), three porcine hemagglutinating encephalomyelitis virus (PHEV) and 18 porcine respiratory coronavirus (PRCV) isolates, mainly from Belgian livestock collected between 2020 and 2023. Spike gene phylogenetic analysis showed nucleotide substitution rates comparable between BCoV and PRCV, while PHEV appeared slower. Unlike severe acute respiratory syndrome coronavirus 2 (SARS-CoV-2), synonymous substitutions were preferred, limiting amino acid variation across decades in the animal coronaviruses. Virus neutralization assays with swine antisera indicated minimal antigenic change in PHEV and PRCV. Recent BCoV isolates showed antigenic divergence from the classical Mebus vaccine strain. The impact of this divergence on vaccine efficacy may warrant further research. Our findings underscore the need for periodic surveillance, as changes in surface proteins may affect pathogenicity, tissue tropism, host range and vaccine efficacy.

## 1. Introduction

Coronaviruses are large positive-sense RNA viruses with 25–31 kb genomes that encode four structural proteins: spike (S), envelope (E), membrane (M) and nucleocapsid (N). They are classified into four genera: alphacoronavirus, betacoronavirus, gammacoronavirus and deltacoronavirus. Betacoronaviruses of subgenus embecovirus, including bovine coronavirus (BCoV) and porcine hemagglutinating encephalomyelitis virus (PHEV), express a fifth structural protein, hemagglutinin esterase (HE) [1].

The coronavirus disease 2019 (COVID-19) pandemic caused by severe acute respiratory syndrome coronavirus type 2 (SARS-CoV-2) highlighted the ability of coronaviruses to generate novel variants through mutation and recombination [2]. This emphasized the need for systematic genomic surveillance and renewed interest in the evolution of other coronaviruses.

The first identification of BCoV was in the United States in 1972. It causes three disease syndromes: calf diarrhea, winter dysentery and respiratory illness [3,4]. No definitive genetic markers distinguish respiratory from enteric strains [5]. Available vaccines include an inactivated Mebus strain vaccine and a live attenuated CA25 strain vaccine. The inactivated vaccine is administered intramuscularly to cows in late gestation to provide passive immunity through colostrum against calf diarrhea [6]. The live attenuated vaccine is administered intranasally to calves for protection against respiratory illness [7]. The first report of PHEV was in Canada in 1957 [8]. Clinical signs including coughing, vomiting, wasting and incoordination may occur in suckling pigs born to naïve sows. Infection is typically subclinical due to maternal immunity [9]. Porcine respiratory coronavirus (PRCV) emerged in Belgium in the early 1980s as a deletion mutant of transmissible gastroenteritis virus (TGEV) [10]. A 207–227 amino acid (aa) deletion in the S protein 5′ region and altered tropism for the respiratory tract distinguish PRCV from TGEV [11]. Infection is mild or subclinical [12]. No commercial vaccines exist for PHEV and PRCV.

The S protein mediates receptor binding and membrane fusion. It is the main target of virus-neutralizing antibodies [13]. The HE acts as a receptor-destroying enzyme, cleaving O-acetylated sialic acids from host cell surfaces to balance S-mediated attachment and release [14]. Antibodies against the HE contribute to virus neutralization, although their effect is weaker than anti-spike responses [15,16]. Mutations in the S protein can enhance infectivity, immune evasion and host adaptation. The HE co-evolves with S, further shaping host adaptation and transmission. Changes in these proteins are central to the emergence of new variants [14,17].

All three coronaviruses are widespread in livestock [9,18,19,20]. Most molecular data focus on North American or Asian strains, with sporadic reports on European strains. This geographic imbalance is notable given the tendency of European strains to diverge from non-European strains. European and non-European BCoV strains are estimated to have diverged in the 1960s to 1970s [21]. Phylogenetic analyses show that European PRCV strains cluster with the TGEV Ia (Miller) genotype, while United States strains cluster with the TGEV Ib (Purdue) and II (variant) genotypes [22].

Mutation analysis indicates that BCoV S protein undergoes diversifying selection on aa residues adjacent to ligand-binding pockets, while the pockets remain under strong negative selection. This pattern preserves essential receptor-binding function while altering adjacent surface-exposed residues. These changes appear driven by immune pressure and can reduce antibody recognition that would block virus attachment through steric hindrance [5]. Emerging PHEV strains appear to cause exclusively respiratory disease, supported by evidence of reduced replication in murine neuronal cells. Loss of neurotropism has been attributed to mutations in the HE lectin binding domain and alternate viral egress pathways [23,24]. An in silico study showed that specific aa mutations in the PRCV S protein receptor binding domain could increase affinity for the human aminopeptidase N receptor [25].

Together, these findings highlight the need for contemporary European data. To address this gap, we analyzed primarily Belgian isolates of BCoV, PHEV and PRCV collected from cattle and pigs with respiratory illness between 2020 and 2023. We characterized genetic diversity in the S and HE proteins relative to historical and vaccine strains and used virus neutralization assays to assess antigenic drift.

## 2. Materials and Methods

### 2.1. Sample Selection

Between 2020 and 2023, viruses were detected in diagnostic samples (nasal swab, deep nasopharyngeal swab and bronchoalveolar lavage samples) collected from cattle and pigs showing signs of respiratory illness. These filtrate samples were mainly from Belgium, but there were also a few from the Netherlands, Germany and Austria (Table 1).

They were submitted to PathoSense, a Ghent University spin-off, or Dierengezondheidszorg Vlaanderen (DGZ), the Flemish animal health service, by veterinarians and farmers for diagnosis by metagenomic analysis [26]. Samples that were positive for at least 1 of the three viruses, BCoV, PHEV and PRCV, were selected for virus isolation on continuous cell lines. A few samples collected from Belgian pig farms as pooled nasal swab samples were included. These samples originated from routine swine influenza monitoring and were submitted directly to our laboratory without screening by metagenomic analysis. They were directly used for virus isolation.

### 2.2. Virus Isolation Assays

The 3 target viruses, BCoV, PHEV and PRCV, were propagated in human rectal tumor 18 (HRT-18), rein de porc diploid (RPD) and swine testis (ST) cell lines, respectively, as previously described [20].

### 2.3. Genetic Characterization

Second passage virus isolates were submitted to PathoSense for whole-genome sequencing using Oxford Nanopore sequencing, on a GridION or PromethION sequencing device (Oxford Nanopore Technologies, Oxford, United Kingdom) and as described previously [27]. Whole-genome de novo assembly was conducted with Canu v2.1.1, followed by polishing with Medaka v1.3.0. The virus genome sequences obtained were deposited in GenBank with the following accession numbers: PX418135-PX418163, PX447284-PX447288, PX580609-PX580612, PX680842 and PX820712 (Supplementary Table S1). Whole genome sequences were aligned by MUSCLE in MEGA version 11, together with sequences in the NCBI GenBank database. The S and HE genes were extracted for phylogenetic analysis. Maximum-likelihood trees were constructed under the GTR+G+I model with 1000 bootstrap replicates.

To estimate evolutionary rates, we retrieved complete S and HE gene sequences from GenBank and aligned them with our study isolates using MUSCLE in MEGA version 11. Sequences that exhibited greater than 99% identity were excluded. For BCoV, only isolates derived from cattle were included (Supplementary Material Table S2). Bayesian evolutionary analyses were conducted in BEAST version 10.5, employing the GTR+G nucleotide substitution model with 4 gamma categories, a strict molecular clock and a coalescent Bayesian Skyride tree prior. Markov Chain Monte Carlo (MCMC) analyses were run for 200 million generations, sampling every 2000 steps. Convergence and effective sample sizes (ESS > 200) were assessed using Tracer version 1.7. Substitution rates are reported as means with 95% highest posterior density (HPD) intervals derived from the posterior distributions.

Site-level selection pressures were assessed using the HyPhy version 2.5.68(MP) package implemented on the Datamonkey adaptive evolution server. To detect episodic diversifying selection, the Mixed Effects Model of Evolution (MEME) version 4.1 was utilized, which identifies individual sites subject to positive selection along a subset of evolutionary branches. Pervasive diversifying selection across the entire phylogeny was evaluated using three complementary methods: Single-Likelihood Ancestor Counting (SLAC) version 2.0, Fixed Effects Likelihood (FEL) version 2.6, and Fast Unconstrained Bayesian AppRoximation (FUBAR) version 2.2. SLAC and FEL infer site-specific selection using count-based and maximum-likelihood approaches, respectively, while FUBAR employs a Bayesian framework to robustly detect weak but consistent selection. Sites were considered under positive selection based on standard significance thresholds: *p* < 0.1 for MEME, FEL, and SLAC, and a posterior probability ≥ 0.9 for FUBAR [28,29,30].

Recombination in full-length S and HE gene alignments was assessed using the RDP5 version 5.64 package, employing seven detection algorithms (RDP, GENECONV, Bootscan, MaxChi, Chimaera, SiScan and 3Seq) [31]. Default settings were used for all methods, and putative recombination events detected by at least four algorithms were considered credible [32].

We estimated the ratio of non-synonymous (amino acid-altering mutations) to synonymous (silent) substitution rates (dN/dS) for the S and HE genes of recent strains with respect to historical reference strains. The reference strains were BCoV Mebus (U00735.2), PHEV VW572 (DQ011855.1) and PRCV 91V44 (OR689864.1). This analysis was performed using MEGA v11 software. Pairwise comparisons of codon sequences were conducted using the Nei–Gojobori method with Jukes–Cantor correction for multiple substitutions. The resulting dN/dS ratios were interpreted as indicators of selective pressure, where values >1 suggest positive selection, ≈1 indicate neutral evolution and <1 imply purifying selection. Amino acid (aa) sequences were also compared for pairwise identity to assess the diversity of recent versus historical isolates. The 3D structures of the S and HE proteins were predicted using AlphaFold 3, and the aa substitutions were mapped onto the structures using ChimeraX version 1.10.1.

### 2.4. Antigenic Characterization

Antigenic variation was assessed by virus neutralization (VN) assays using polyclonal swine antisera raised against historical and recent strains. For each virus, 2 antisera were produced in swine using the protocol previously described [20]. For BCoV, the Mebus strain (USA, 1972) and a 2020 isolate from Belgium (BCoV-Gent/PS-666/2020) were used. For PHEV, the VW572 strain (Belgium, 1972) and PHEV-Gent/PS-412/2020 (The Netherlands, 2020) were selected. For PRCV, Belgian isolates 91V44 (1991) and PRCV-Gent/PS-071/2020 (2020) were included. The VN assays for the three viruses were performed as previously described [20]. All assays began with antisera diluted 1/4. Final VN titers were expressed as the reciprocal of the highest serum dilution that neutralized 50% of 100 TCID_50_ of virus.

## 3. Results

### 3.1. Genetic Characterization

#### 3.1.1. Bovine Coronavirus

**Figure 1:**
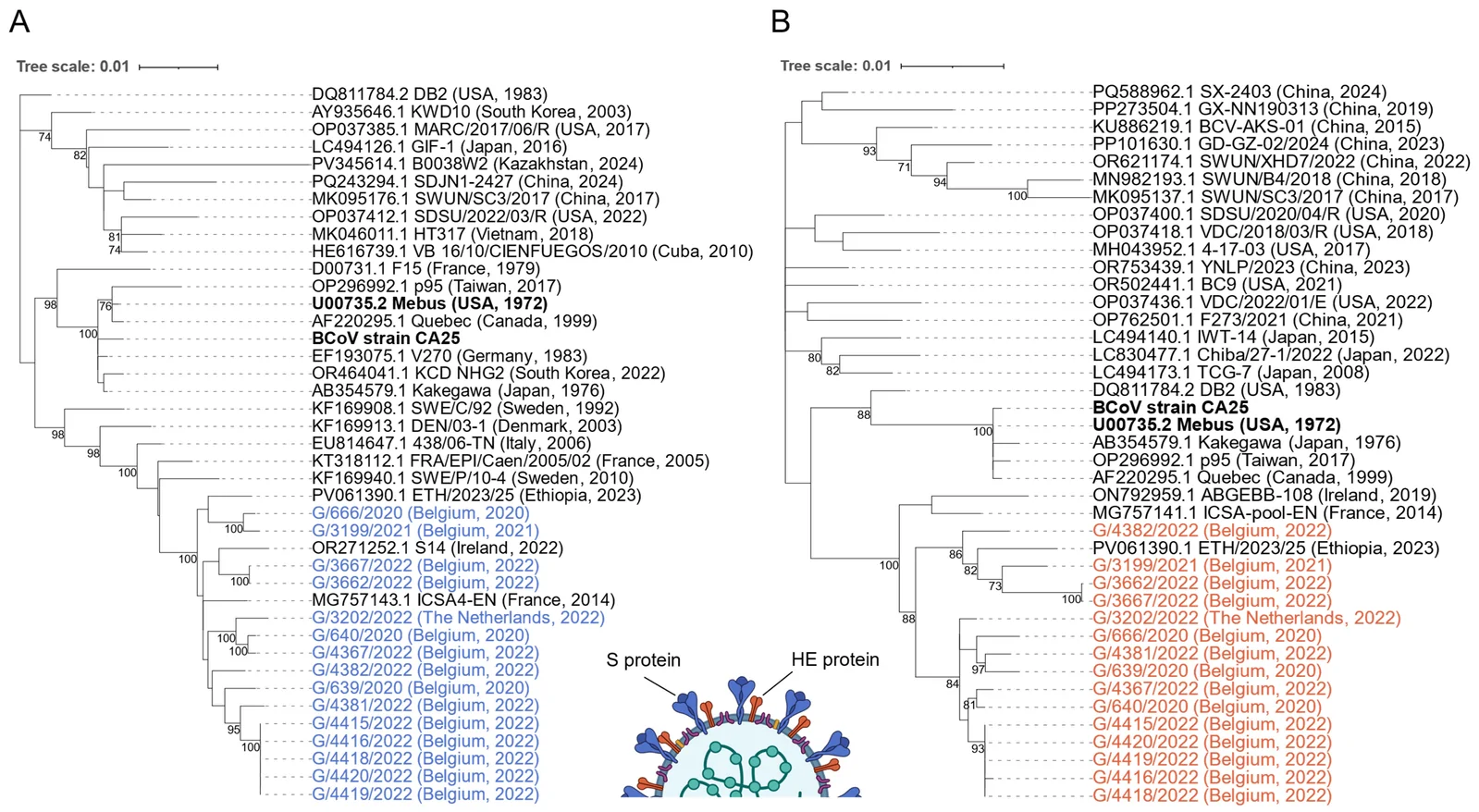
Maximum-likelihood phylogenetic trees of the S (**A**) and HE (**B**) genes of BCoV isolates. Trees were generated using MEGA v11 with the GTR+G+I model and 1000 bootstraps. The vaccine strains are shown in bold. The isolates from this study are shown in blue (**A**) and orange (**B**) text. Gene sequences obtained from the GenBank database are in black text. Bootstrap support values are indicated at the nodes. The scale bars above the trees indicate the number of nucleotide substitutions per site.

Phylogenetic analysis of S and HE genes showed clear geographical clustering, with American and Asian strains grouping separately from European isolates (Figure 1). Our recent isolates clustered together with other recent European isolates but separately from the historical European strains F15 and V270 (nucleotide identity of S protein 96.5–97.2%). An isolate from Ethiopia (PV061390) also clustered together with the recent isolates from Europe (nucleotide identity of S gene 98.6–99.0%; HE gene 97.8–98.8%).

Bayesian analysis estimated the evolutionary rate of the S gene at 6.64 × 10^−4^ (95% HPD: 5.78 × 10^−4^–7.50 × 10^−4^) nucleotide substitutions/site/year. Analysis of the S gene revealed a low global dN/dS ratio across all utilized site-selection models (ranging from 0.1852 to 0.2342), indicating that the gene is predominantly subjected to strong purifying selection (Table 2). Similarly, the pairwise dN/dS ratios of the recent isolates relative to the Mebus strain ranged from 0.19 to 0.22 for the S gene (Supplementary Material Table S3). Despite this gene-wide constraint, evidence of site-specific diversifying selection was observed. The episodic model (MEME) identified 57 sites under positive selection along a subset of evolutionary branches. Fewer sites were implicated under pervasive selection across the entire phylogeny, with 24, 25, and 15 sites identified by SLAC, FEL, and FUBAR, respectively. Cross-method comparison revealed a robust consensus profile, with 10 key codons (12, 113, 121, 146, 499, 501, 509, 514, 927, and 1237) simultaneously identified by multiple pervasive selection algorithms, indicating ongoing, uniform evolutionary pressure at these positions. Notably, several identified sites correspond to critical functional domains of the S protein, reflecting targeted adaptive evolution amidst a highly conserved structural backbone.

The HE gene has evolved more slowly, at 1.78 × 10^−4^ nucleotide substitutions/site/year (95% HPD: 1.23 × 10^−7^–3.24 × 10^−4^). The broad HPD interval may reflect low sequence diversity or also the presence of rare events such as the insertion of 12 nucleotides in some strains from the United States and China. Like the S gene, the HE gene global dN/dS ratio remained consistently low across the counting and likelihood-based models (0.1906 for MEME, 0.2573 for SLAC, and 0.2396 for FEL), indicating that the gene is evolving under overall strong purifying selection. In line with this, the pairwise dS/dN ratios of the HE gene of recent isolates with respect to the Mebus strain ranged from 0.10 to 0.20. Despite this, a small number of codons showed evidence of diversifying selection. The episodic model (MEME) identified four sites (65, 181, 243, and 309) experiencing selection along a subset of evolutionary branches. Under pervasive selection frameworks, SLAC and FEL each identified a single codon under positive selection (codon 66), while the highly sensitive FUBAR method identified four sites (66, 147, 218, and 380). Notably, codon 66 was robustly supported as a target of pervasive diversifying selection by all three global models (SLAC, FEL, and FUBAR). This suggests potential evolutionary significance of the site in the long-term adaptation of the BCoV HE protein.

In line with the demonstrated strong purifying selection, amino acid sequence identity was high across geographical regions. Compared with classical strains (Kakegawa, Mebus, Quebec), recent European isolates showed 96.0–96.8% S aa sequence identity. The recent European strains also showed similar diversity with the S protein of the BCoV vaccine strain CA25 (aa sequence identity of 95.9–96.3%). Meanwhile, the aa sequence identity among recent European strains ranged from 98.5 to 100% (Table 3). Individual sequence identities are shown in Supplementary Material Figure S1A,B.

**Figure 2:**
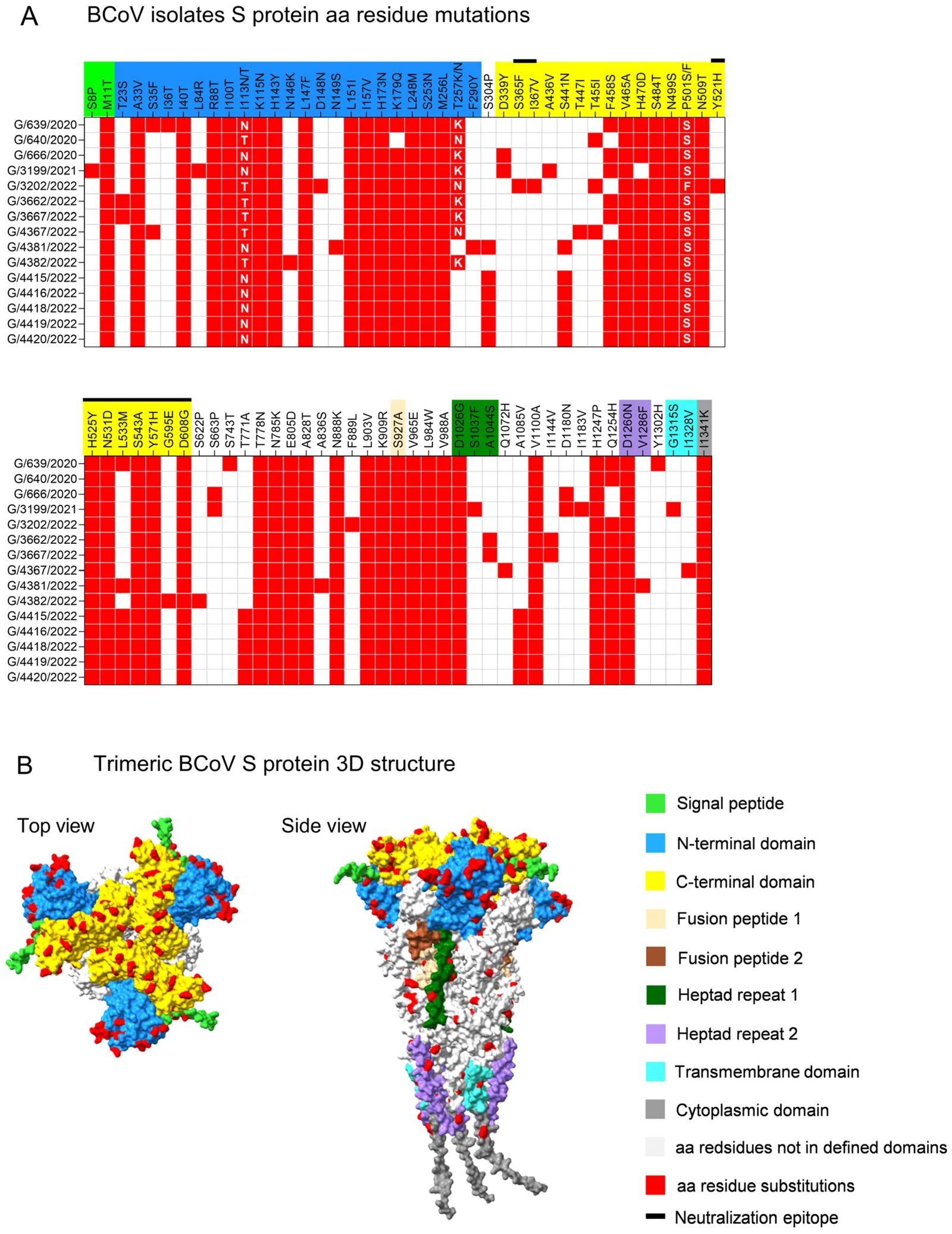
aa substitutions in the BCoV S protein of recent isolates, shown as (**A**) a heatmap and (**B**) locations on the predicted trimeric 3D structure relative to the Mebus strain. The 3D structures were predicted using AlphaFold3 and annotated with ChimeraX. Rows in the heatmap represent individual isolates, and columns correspond to specific aa positions. Red boxes indicate the presence of a substitution; white boxes indicate its absence. Specific substitutions are noted when multiple variants occur at the same position.

**Figure 3:**
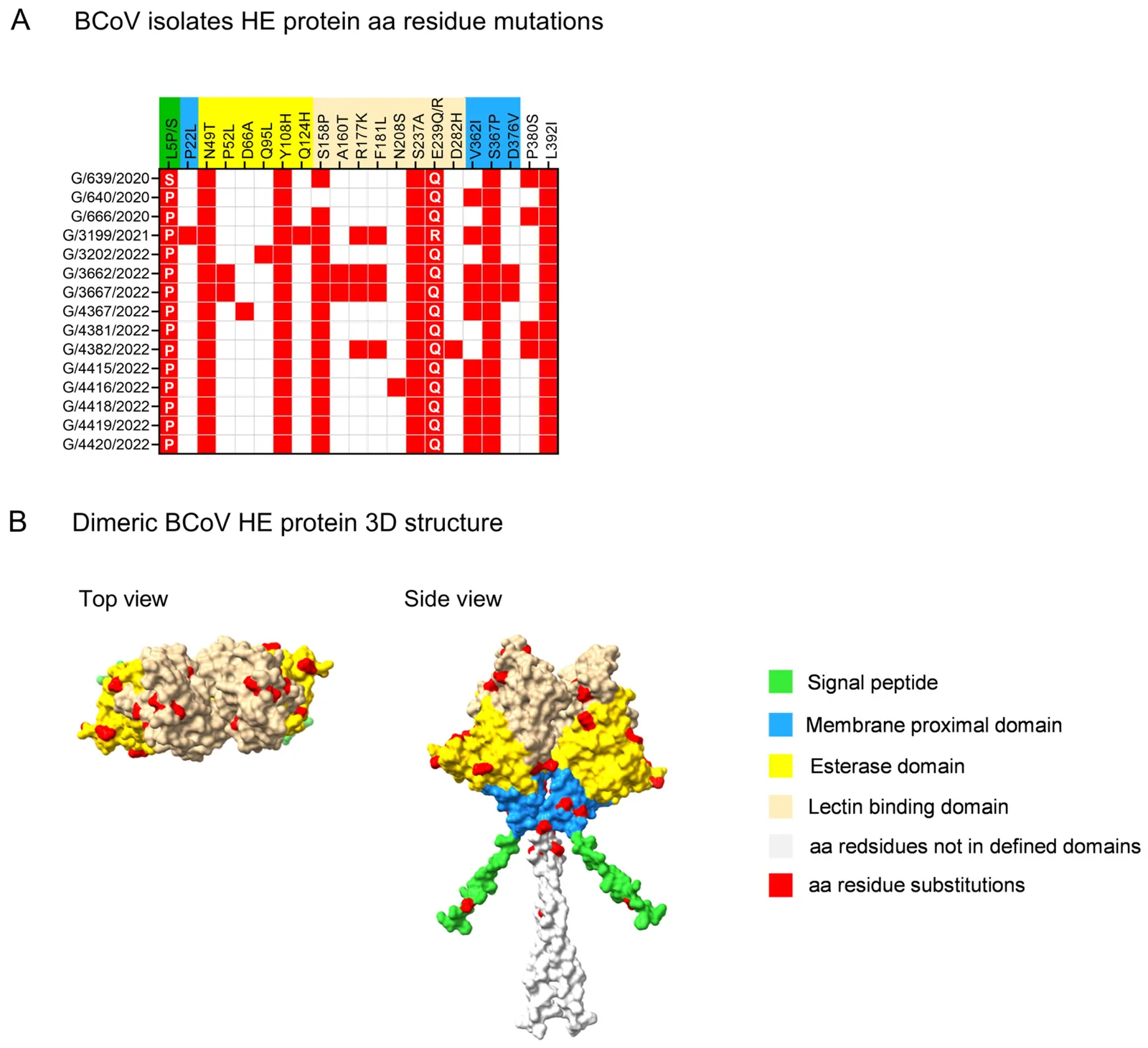
aa substitutions in the BCoV HE protein of recent isolates, shown as (**A**) a heatmap and (**B**) locations on the predicted trimeric 3D structure relative to the Mebus strain. The 3D structures were predicted using AlphaFold3 and annotated with ChimeraX. Rows in the heatmap represent individual isolates, and columns correspond to specific aa positions. Red boxes indicate the presence of a substitution; white boxes indicate its absence. Specific substitutions are noted when multiple variants occur at the same position.

Analysis of neutralization epitopes revealed mutations in both domains I and II of the S protein. In domain I, isolate G/3202/2022, which carried S365F and I367V relative to the Mebus strain (Figure 2). In domain II, all study isolates shared substitutions H525Y, N531D, S543A, Y571H, and D608G, with additional changes Y521H, L533M, and G594E in some strains. Within the HE lectin binding domain, mutations S158P, S237A, and E239Q were present in most isolates (Figure 3).

#### 3.1.2. Porcine Hemagglutinating Encephalomyelitis Virus

**Figure 4:**
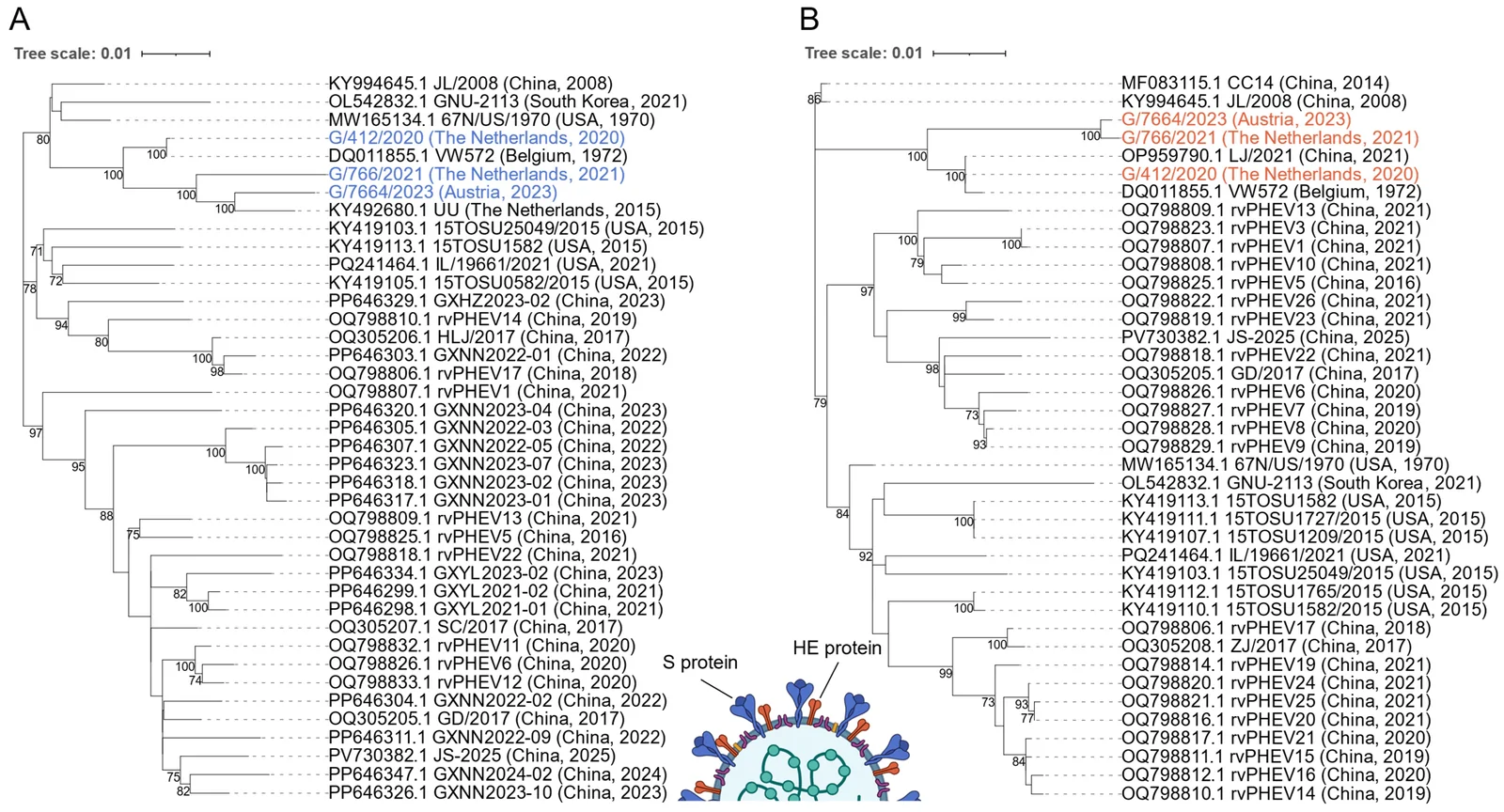
Maximum-likelihood phylogenetic tree of the S (**A**) and HE (**B**) gene sequences of PHEV isolates. The trees were generated using MEGA v11 with the GTR+G+I model and 1000 bootstraps. The isolates from this study are shown in blue (**A**) and orange (**B**) text. Gene sequences obtained from the GenBank database are in black text. Bootstrap support values are indicated at the nodes. The scale bar above the trees indicates the number of nucleotide substitutions per site.

Phylogenetic analysis of the PHEV S gene showed European isolates forming a distinct clade from Asian and American isolates (nucleotide identity with American isolates 95.1–97.9%; Asian isolates 94.2–98.0%) (Figure 4). For the HE gene, European isolates cluster separately from those of American origin (nucleotide identity 94.0–97.3%). The HE of an isolate from China (OP959790) also clustered together with the recent isolates from Europe (nucleotide identity 96.9–100%). This strain is almost genetically identical to the historical Belgian strain VW572. This suggests a shared ancestry between this Chinese strain and European strains.

The evolutionary rate of the S gene was 1.72 × 10^−4^ substitutions/site/year (95% HPD: 1.01 × 10^−4^–2.49 × 10^−4^). Selection pressure analysis on the S gene revealed a consistent trend of gene-wide constraint, with global dN/dS ratios ranging from 0.2415 (MEME and FEL) to 0.2544 (SLAC), indicating predominant purifying selection (Table 4). In line with this, the pairwise dN/dS ratios of the recent sequences with reference to VW572 ranged from 0.16 to 0.25 for S gene (Supplementary Material Table S3). Site-specific analyses identified codons actively evolving under diversifying selection. The episodic model (MEME) identified 16 positively selected sites along specific evolutionary lineages. Pervasive selection analysis identified nine, 13, and 18 sites using SLAC, FEL, and FUBAR, respectively. Cross-method comparison revealed a highly robust consensus selection profile across the entire phylogeny. Specifically, six key codons (11, 25, 154, 770, 1012, and 1330) were simultaneously identified by all four analytical frameworks, marking them as major hotspots for long-term adaptive evolution. Additionally, sites 696 and 842 demonstrated strong multi-method support, being recognized by at least two pervasive selection models alongside individual episodic or Bayesian detections.

The HE gene evolved at 2.70 × 10^−4^ substitutions/site/year (95% HPD: 1.70 × 10^−4^–3.70 × 10^−4^). The availability of fewer sequences for the HE gene analysis may have resulted in an impression of a similar evolution rate to the S gene. The global dN/dS ratios across the counting and likelihood-based models ranged from 0.2311 (MEME) to 0.2866 (SLAC), reflecting a dominant regime of purifying selection across the gene as a whole. The pairwise dN/dS ratios of the H gene of recent isolates were also low, ranging from 0.26 to 0.30. Under this conservative background, site-specific analyses revealed a highly localized and largely episodic pattern of diversifying selection. The episodic framework (MEME) identified five codons under selection along specific evolutionary lineages. Conversely, the conservative SLAC method detected zero sites under pervasive selection, while FEL and FUBAR identified three sites (24, 160, and 411) and one site (411), respectively. Cross-method comparison highlighted codon 411 as a critical evolutionary hotspot, as it was simultaneously identified by three independent frameworks (MEME, FEL, and FUBAR). Additionally, site 160 demonstrated strong multi-method support, being recognized by both the episodic (MEME) and maximum-likelihood pervasive (FEL) models, pointing to its potential role in targeted viral adaptation.

**Figure 5:**
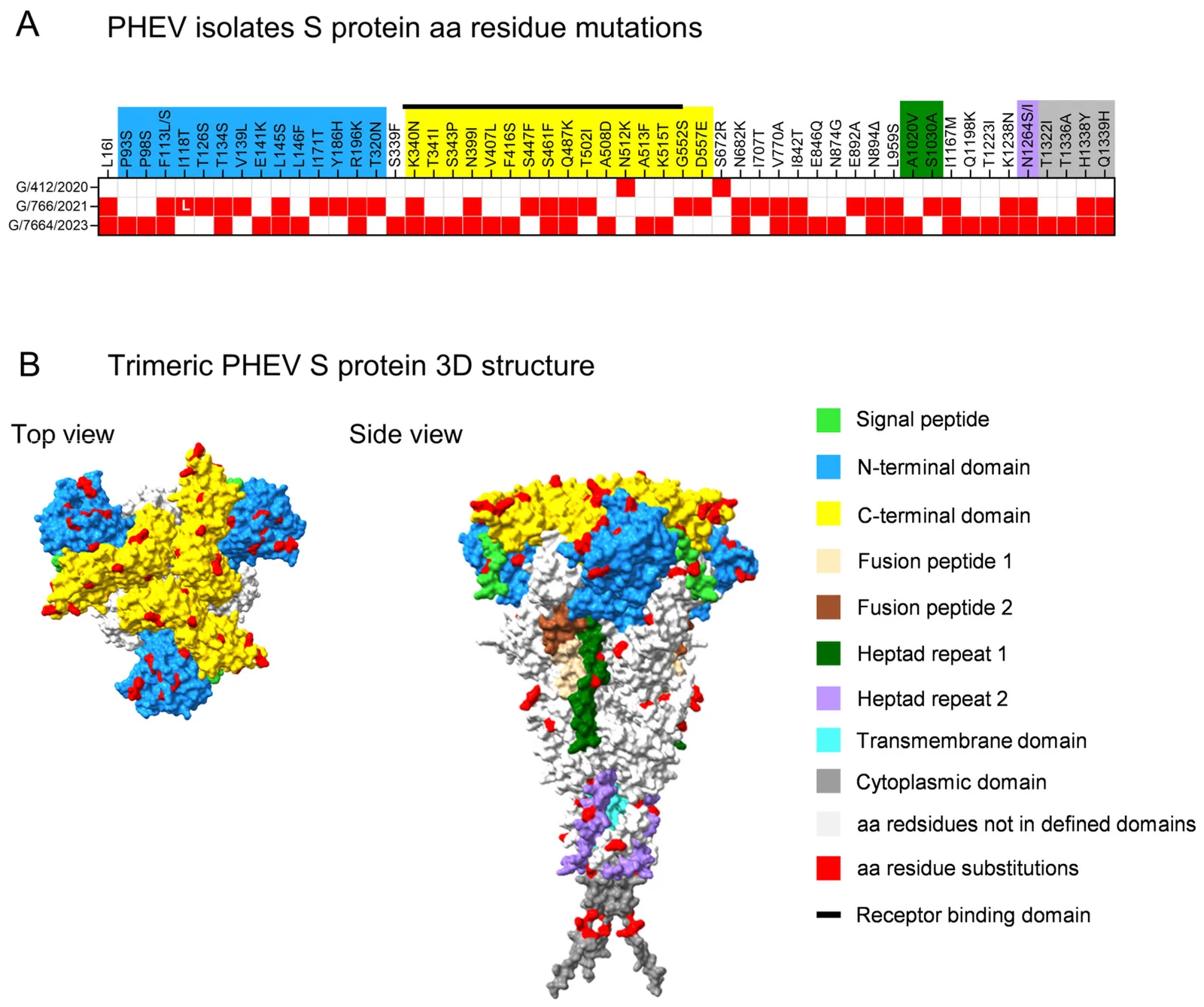
aa substitutions in the PHEV S protein of recent isolates, shown as (**A**) a heatmap and (**B**) locations on the predicted trimeric 3D structure relative to the VW572 strain. The 3D structures were predicted using AlphaFold3 and annotated with ChimeraX. Rows in the heatmap represent individual isolates, and columns correspond to specific aa positions. The Δ at position 894 in the S protein denotes a deletion. Red boxes indicate the presence of a substitution; white boxes indicate its absence. Specific substitutions are noted when multiple variants occur at the same position.

**Figure 6:**
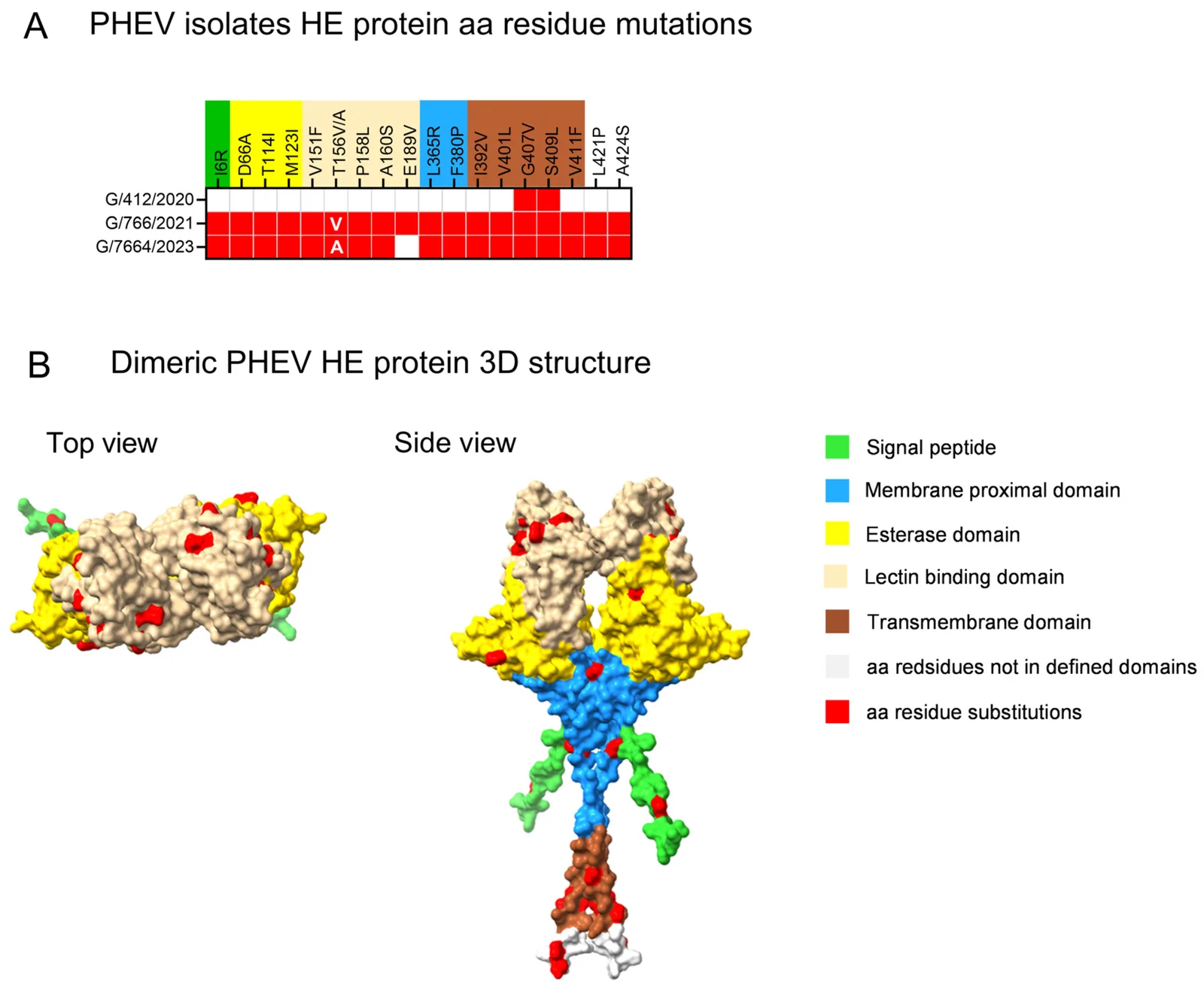
aa substitutions in the PHEV HE protein of recent isolates, shown as (**A**) a heatmap and (**B**) locations on the predicted trimeric 3D structure relative to the VW572 strain. The 3D structures were predicted using AlphaFold3 and annotated with ChimeraX. Rows in the heatmap represent individual isolates, and columns correspond to specific aa positions. Red boxes indicate the presence of a substitution; white boxes indicate its absence. Specific substitutions are noted when multiple variants occur at the same position.

For isolate G/412/2020, no synonymous substitutions were detected, preventing calculation of dN/dS, likely due to its high sequence identity with the VW572 reference strain. Sequence identity between recent isolates and historical strains (67N, VW572) was high (93.9–99.9%), with slightly more divergence from Asian and North American strains (92.5–100%) (Table 2). Individual sequence identities are shown in Supplementary Material Figure S1. Within the receptor binding domain (RBD) of the S protein (aa 311–608), isolate G/766/2021 carried eight substitutions and G/7664/2023 carried eleven compared to VW572, while G/412/2020 had only one (Figure 5). In the HE lectin binding domain, substitutions V151F, P158L, and A160S were detected in both G/766/2021 and G/7664/2023 (Figure 6).

#### 3.1.3. Porcine Respiratory Coronavirus

**Figure 7:**
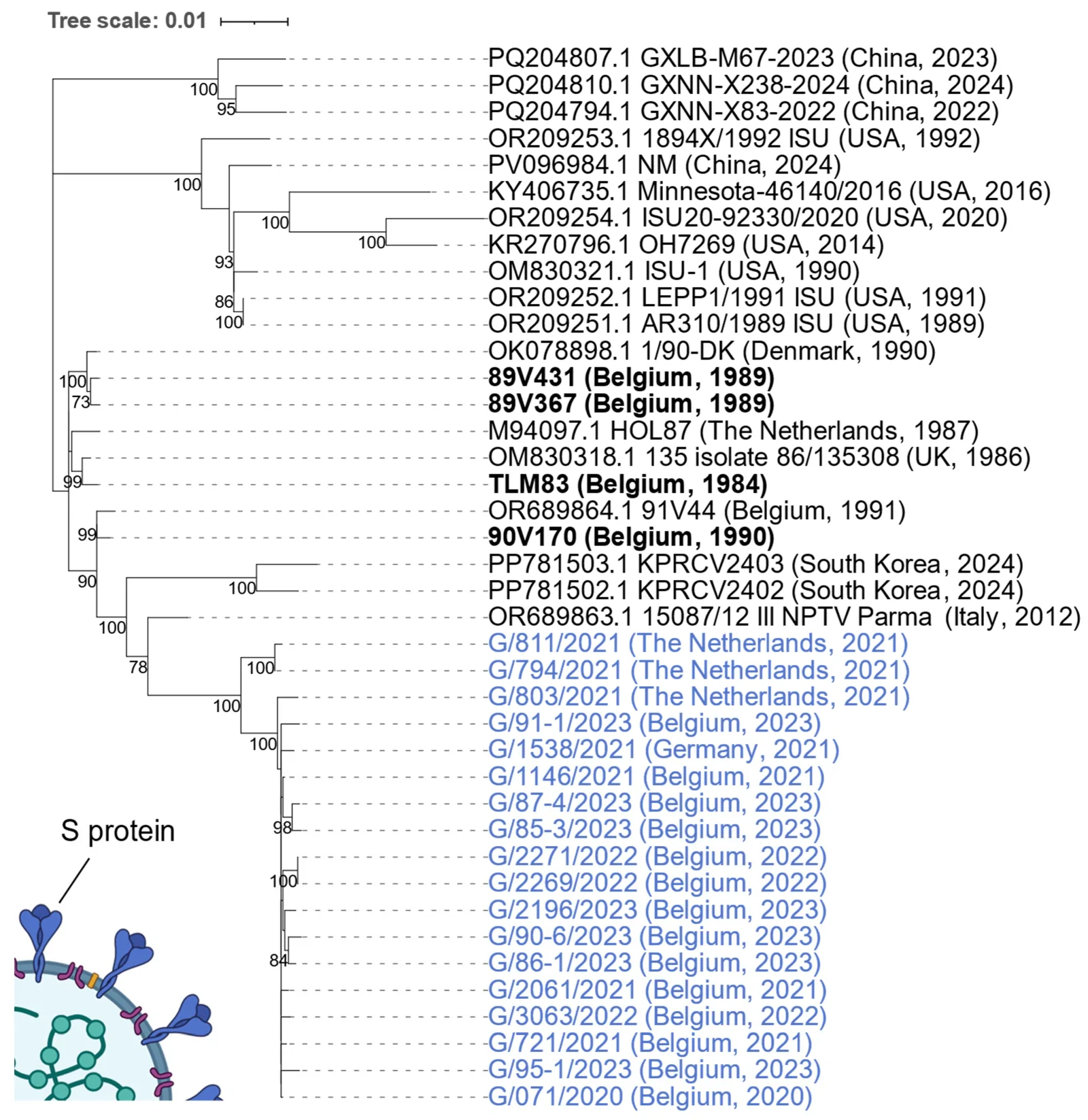
Maximum-likelihood phylogenetic tree of the S gene sequences of PRCV isolates. The tree was generated using MEGA v11 with the GTR+G+I model and 1000 bootstraps. The isolates obtained in this study are shown in blue text. Isolates from archived samples are shown in bold. Gene sequences obtained from the GenBank database are in black text. Bootstrap support values are indicated at the nodes. The scale bar above the tree indicates the number of nucleotide substitutions per site.

The maximum likelihood phylogeny of PRCV S genes showed separate clades for isolates from China and the United States from those in Europe and South Korea (Figure 7). Furthermore, the historical European strains tend to cluster in a distinct clade from the more recently circulating strains.

The evolutionary rate of the S gene was 7.62 × 10^−4^ substitutions/site/year (95% HPD: 6.79 × 10^−4^–8.47 × 10^−4^). Analysis of the PRCV S gene demonstrated a highly constrained evolutionary profile at the global level, with cumulative dN/dS ratios consistently low across the models (0.2091 for MEME and FEL, and 0.2339 for SLAC), confirming strong gene-wide purifying selection (Table 5). Consistent with this, the dN/dS ratios for recent isolates with respect to the PRCV 91V44 isolate ranged from 0.14 to 0.17 (Supplementary Material Table S3). Despite this structural conservation, site-specific evaluations revealed a distinct pattern of highly localized and primarily episodic adaptive evolution. The episodic model (MEME) identified 15 codons under positive selection along specific evolutionary lineages. In contrast, the highly conservative SLAC model identified zero sites under pervasive selection across the entire phylogeny. However, the alternative pervasive frameworks provided targeted support: FEL identified a single site (180), while the Bayesian FUBAR method identified four sites (22, 396, 621, and 753). Cross-method comparisons highlighted several codons with multi-method validation, most notably sites 22, 180, 621, and 753, which were simultaneously flagged by both episodic (MEME) and pervasive (FEL or FUBAR) selection algorithms, pointing to their potential functional significance in PRCV S adaptation.

**Figure 8:**
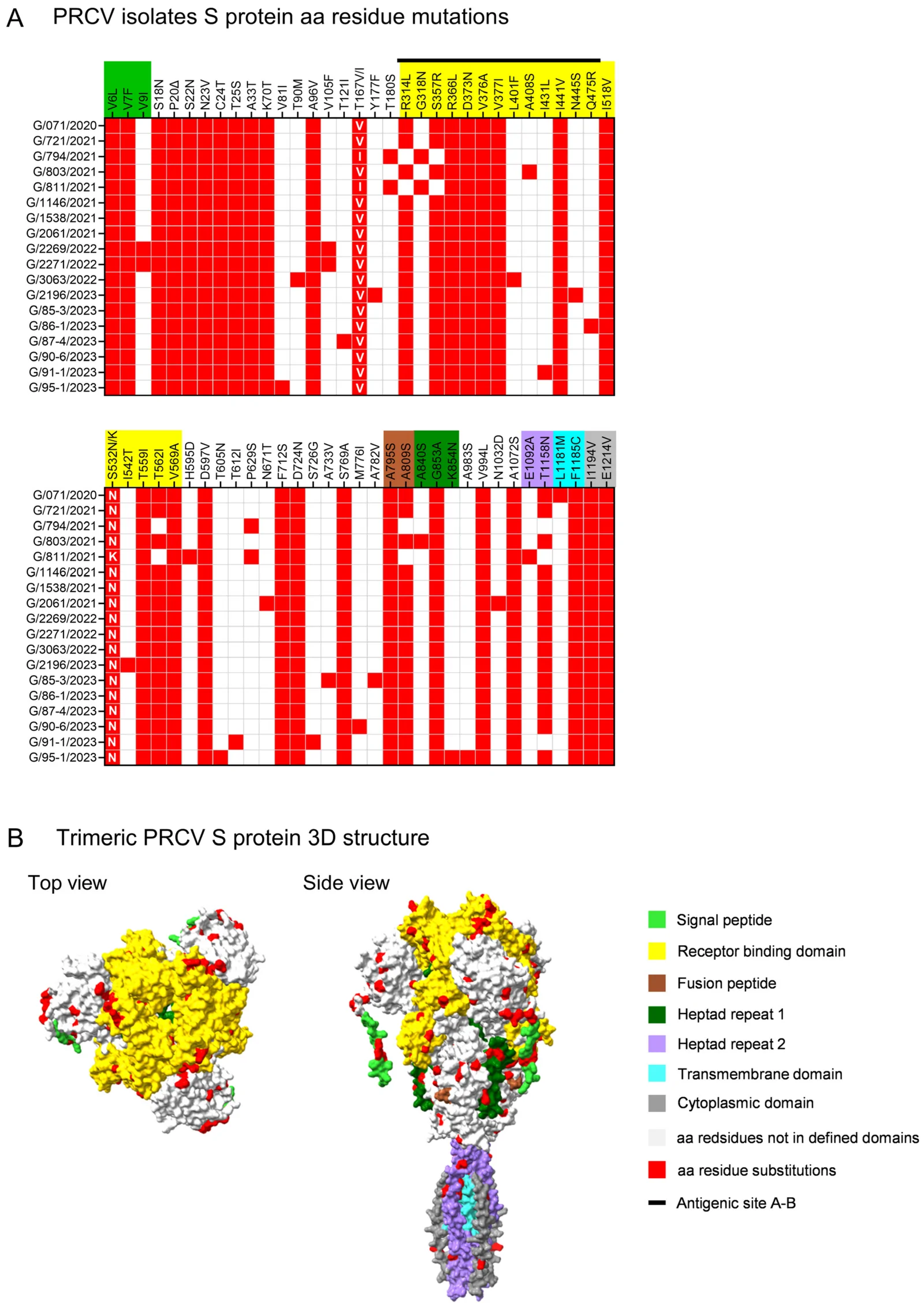
aa substitutions in the PRCV S protein of recent isolates, shown as (**A**) a heatmap and (**B**) locations on the predicted trimeric 3D structure relative to the PRCV isolate 91V44. The 3D structures were predicted using AlphaFold3 and annotated with ChimeraX. Rows in the heatmap represent individual isolates, and columns correspond to specific aa positions. The Δ at position 20 in the S protein denotes a deletion. Red boxes indicate the presence of a substitution; white boxes indicate its absence. Specific substitutions are noted when multiple variants occur at the same position.

Amino acid sequence identity was lowest between European and U.S. strains (93.3–95.6%) (Table 3). Comparisons with classical European strains showed 96.5–97.0% identity, while identity with other European isolates ranged from 96.6 to 98.2%. The highest similarity was observed among the recent isolates (98.7–100%). Individual sequence identities are shown in Supplementary Material Figure S1. Within the receptor binding domain, substitutions included I441V, I518V, S532N, T559I, T562I, and V569A (Figure 8).

Recombination screening of the full-length S and HE gene alignments flagged isolated putative breakpoints. However, none of the signals were supported by at least four methods, which was the predefined threshold for considering an event credible. As a result, there were no recombination events detected in the S and HE genes of recent BCoV and PHEV isolates or in the S genes of PRCV isolates.

### 3.2. Antigenic Characterization

#### 3.2.1. Bovine Coronavirus

**Figure 9:**
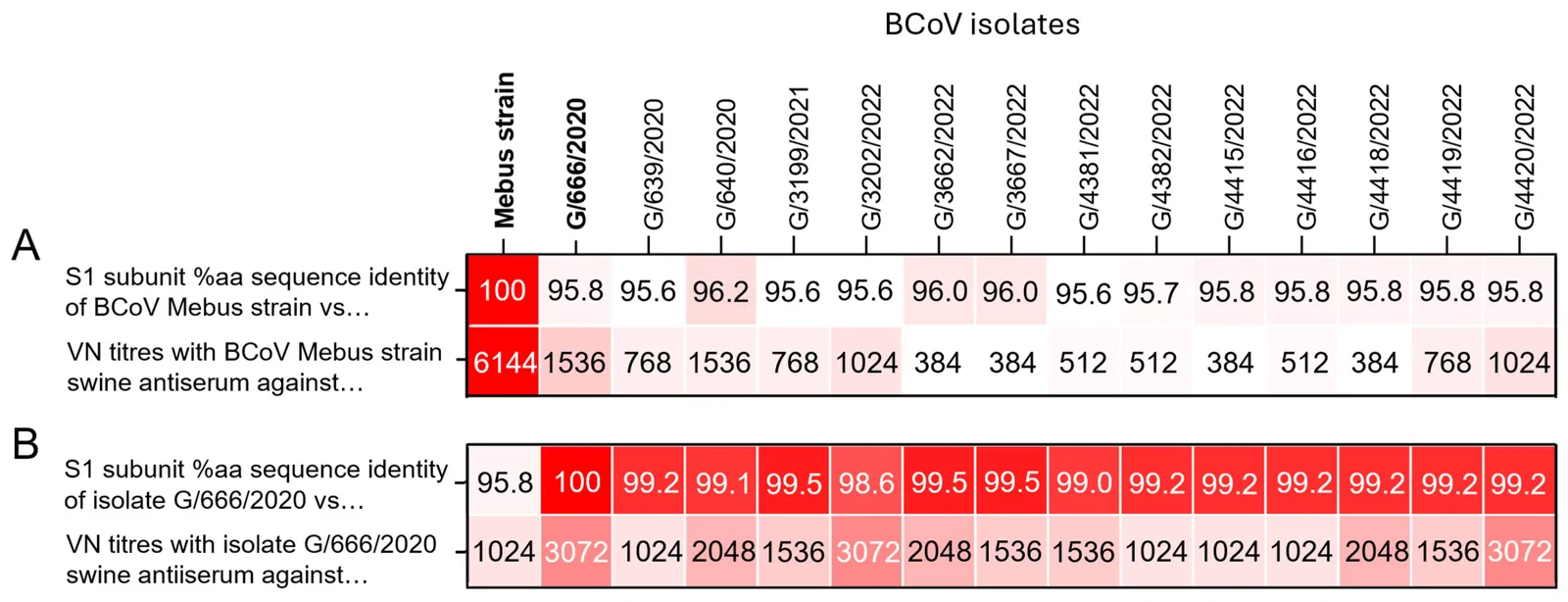
Genetic relatedness and cross-neutralization patterns between BCoV Mebus reference strain and recent isolates. Heatmaps show S1 percent aa identity and corresponding VN titers. Color intensity reflects the magnitude of sequence identity or VN titer. Panel (**A**) compares contemporary isolates with the Mebus reference strain. The upper row presents S1 amino-acid identity values, and the lower row shows VN titers generated using hyperimmune swine antisera raised against Mebus. Panel (**B**) compares the same isolates with the field strain G/666/2020. The upper row displays S1 percent aa identity values relative to G/666/2020, and the lower row shows VN titers obtained using antisera raised against this isolate.

Virus neutralization assays revealed reduced cross-reactivity between historical and recent strains. Using antisera against the Mebus strain, titers against heterologous strains were reduced 4- to 16-fold compared with homologous virus (Figure 9). In contrast, antisera raised against the recent isolate G/666/2020 showed less than a 4-fold reduction across strains, including Mebus.

#### 3.2.2. Porcine Hemagglutinating Encephalomyelitis Virus

**Figure 10:**
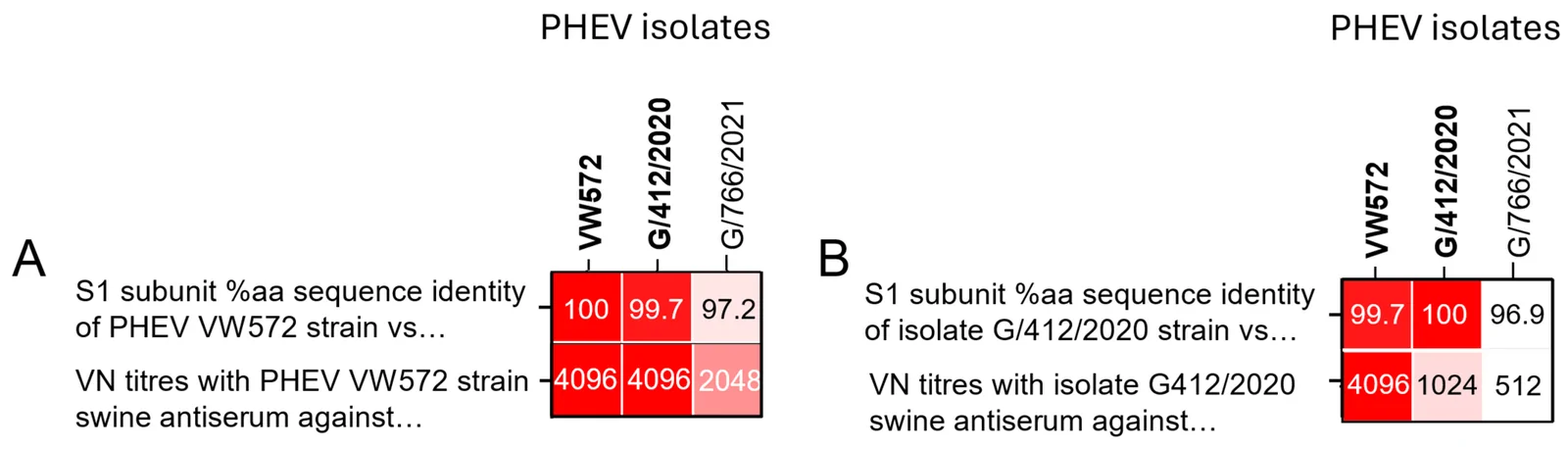
Genetic relatedness and cross-neutralization patterns between PHEV VW572 reference strain and recent isolates. Heatmaps show S1 percent aa identity and corresponding VN titers. Color intensity reflects the magnitude of sequence identity or VN titer. Panel (**A**) compares contemporary isolates with the PHEV VW572 reference strain. The upper row presents S1 amino acid identity values, and the lower row shows VN titers generated using hyperimmune swine antisera raised against Mebus. Panel (**B**) compares the same isolates with the field strain G/412/2020. The upper row displays S1 percent aa identity values relative to G/412/2020, and the lower row shows VN titers obtained using antisera raised against this isolate.

No significant antigenic differences were detected among PHEV isolates. Neutralization titers using antisera against either the historical VW572 strain or the recent G/412/2020 isolate differed by less than 4-fold (Figure 10).

#### 3.2.3. Porcine Respiratory Coronavirus

**Figure 11:**
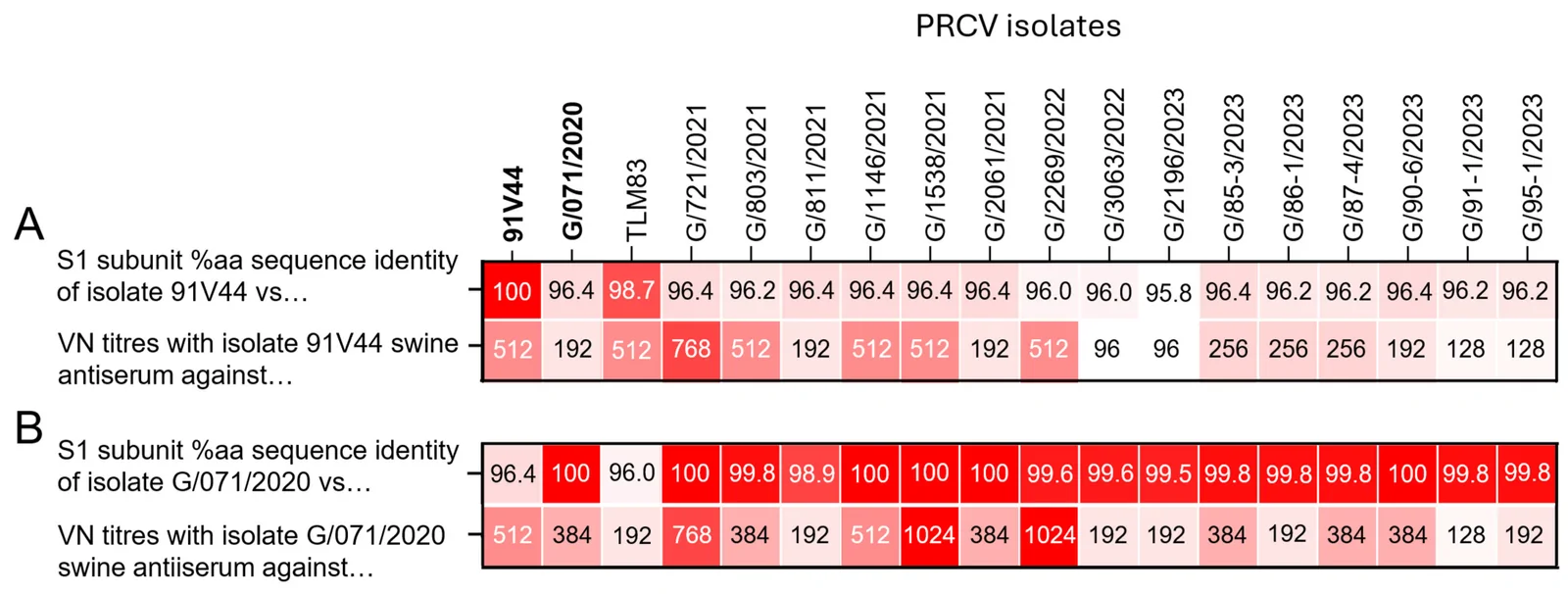
Genetic relatedness and cross-neutralization patterns between PRCV isolate 91V44 reference strain and recent isolates. Heatmaps show S1 percent aa identity and corresponding VN titers. Color intensity reflects the magnitude of sequence identity or VN titer. Panel (**A**) compares contemporary isolates with the PRCV isolate 91V44 reference strain. The upper row presents S1 amino acid identity values, and the lower row shows VN titers generated using hyperimmune swine antisera raised against Mebus. Panel (**B**) compares the same isolates with the field strain G/071/2020. The upper row displays S1 percent aa identity values relative to G/071/2020, and the lower row shows VN titers obtained using antisera raised against this isolate.

Neutralization assays for PRCV showed modest variation. Antisera against historical isolate 91V44 exhibited 0.7- to 4-fold variation in titer against heterologous strains. Antisera against the recent isolate G/071/2020 showed smaller differences (0.4- to 2-fold) across heterologous strains (Figure 11).

## 4. Discussion

This study describes the genetic and antigenic diversity of the S and HE surface proteins of recent western European BCoV, PHEV and PRCV isolates. Phylogenetic analyses showed that our BCoV, PHEV, and PRCV isolates from 2020–2023 clustered with other European strains, forming clades distinct from American strains. These findings confirm earlier reports of geographical clustering in BCoV, PHEV and PRCV [33,34]. This pattern likely reflects the predominance of regional livestock trade within Europe over intercontinental movement [35]. This consistent clustering supports the idea of long-term regional evolution and limited intercontinental transmission.

The nucleotide substitutions rates calculated for the BCoV S gene were comparable with those reported for human betacoronaviruses HCoV-HKU1, HCoV-OC43, MERS-CoV and SARS-CoV-2 [36,37,38]. The estimated evolutionary rate for the PHEV S gene was about 4-fold lower than that of the SARS-CoV-2 S gene [38]. However, the evolutionary rate of the PHEV S gene may be an underestimation due to the geographical bias of the S gene sequences available for analysis in the GenBank database. When we attempted a sensitivity analysis by removing sequences from the over-represented region, the remaining dataset lacked sufficient temporal data for reliable molecular-clock inference. This outcome highlights that current PHEV genomic data is too sparse to allow more robust assessment of evolutionary dynamics. Broader and more geographically diverse sequencing efforts will be essential for refining rate estimates for this virus. For the alphacoronavirus PRCV, the S gene evolutionary rate was comparable to HCoV 229E and SARS-CoV-2 but 2-fold higher than HCoV NL63 [38,39,40]. Compared to another porcine alphacoronavirus, porcine deltacoronavirus, the mean evolutionary rate of the S gene of PRCV was 2-fold lower [41].

Across all three coronaviruses examined, BCoV, PHEV, and PRCV, the S gene exhibited a dominant pattern of strong purifying selection, punctuated by episodic diversifying selection at a limited number of codons. The HE gene, evaluated for the betacoronaviruses BCoV and PHEV, demonstrated a similarly conserved profile. The highest number of positively selected sites were detected in the BCoV S gene. Analysis using MEME identified 57 codons under episodic selection and SLAC/FEL/FUBAR consistently supported a core subset (codons 12, 113, 121, 146, 499, 501, 509, 514, 927, 1237, 1362). This pattern of episodic selection being concentrated in the S1 subunit aligns with previous analyses of BCoV and HCoV OC43, associated with antigenic drift and adaptation to respiratory or enteric tissues [42,43]. The HE gene of BCoV, by contrast, showed only 4–5 positively selected sites, consistent with earlier findings that HE evolves more slowly due to its structural constraints and secondary role in receptor interaction [44].

PHEV showed a moderate number of positively selected sites in the S gene with 16–18 sites across methods, with several codons (11, 25, 154, 770, 842, 1012, 1244, 1330) detected by at least two methods. This suggests localized adaptive pressures. These sites also cluster primarily in the S1 subunit, echoing patterns described for other neurotropic betacoronaviruses, such as murine hepatitis virus, where selection is thought to reflect host immune pressure and tissue tropism shifts [45,46]. The PHEV HE gene showed minimal evidence of diversifying selection (0–5 sites), consistent with the conserved nature of HE in lineage A betacoronaviruses. Pairwise comparisons revealed a higher dN/dS ratio in the PHEV HE gene than in BCoV. Rather than a higher rate of adaptive evolution, this trend likely reflects our limited sequence diversity. In datasets with few synonymous substitutions and short branch lengths, minor alignment changes can inflate dN/dS ratios.

PRCV exhibited the lowest level of diversification. Using MEME, 15 codons were detected under selection, while only four sites were supported by FUBAR and FEL identified one site (codon 180). The absence of SLAC-supported sites and the low dN/dS ratio (0.2091) suggest that PRCV S is under stronger functional constraint than BCoV or PHEV. This is consistent with earlier PRCV/TGEV evolutionary studies showing high conservation of S, particularly in regions associated with altered tropism following the deletion in the N-terminal domain [11,47]. The few sites detected across multiple methods (22, 180, 621, 753) may represent focal points of adaptation linked to receptor interaction or immune evasion, but the overall pattern suggests limited ongoing diversification.

During the COVID-19 pandemic, SARS-CoV-2 showed dN/dS ratios >1, as it accumulated mutations to adapt to the human host [48]. In contrast, the three animal coronaviruses examined here are already well adapted to their respective hosts. Consequently, they require fewer aa substitutions to maintain transmission. This was reflected in the low pairwise dN/dS ratios of our isolates with respect to the reference strains. Despite this overall genetic stability, our virus neutralization assays showed antigenic drift in recent BCoV isolates when tested against BCoV Mebus strain antiserum. Two major conformational neutralization domains have previously been described on the BCoV S protein. Domain I (aa residues 351–403) and domain II (aa residues 517–621) [49]. In our study, multiple mutations were localized within these precise regions. Notably, the consistently observed substitutions H525Y, N531D, S543A, Y571H and D698G fall within domain II. Further functional assays would be required to determine if these specific substitutions observed in our study facilitate immune evasion. Individual mutations in domain II can alter monoclonal antibody binding [49].

Widespread vaccination has been associated with the development of immune evasion mutations in SARS-CoV-2 [50]. It is therefore possible that vaccine pressure from the Mebus strain-based BCoV vaccine may have had an influence on the development of antigenic drift. However, this is difficult to assess due to limited vaccine coverage data. The inactivated BCoV vaccine formulated with the classical Mebus strain relies on humoral immunity and is primarily administered to pregnant cows to provide passive protection during the early neonatal period. This vaccine targets neonatal calf diarrhea. A recently launched live attenuated BCoV vaccine based on the CA25 strain induces strong mucosal and cellular immunity by replicating locally in the upper respiratory tract, enabling protection even in colostrum-deprived neonatal calves [7]. Information on the geographical origin and year of isolation of the CA25 vaccine strain is not publicly available. We did however determine that its S protein shares a 98.5% aa sequence identity with the Mebus strain. The observed antigenic divergence from the Mebus strain suggests potential implications for vaccine performance. However, our study did not include challenge experiments or field vaccine-response data. Therefore, no conclusions regarding in vivo vaccine efficacy can be drawn. Previous studies show both these vaccines are still beneficial in European cattle [6,7]. Our results, however, suggest monitoring the protective efficacy of currently available BCoV vaccines may be warranted.

No significant antigenic differences were detected between historic and recent isolates of PHEV and PRCV, where titers differed by less than 4-fold. There was no clear relationship between PRCV S protein aa identity and swine antisera neutralizing activity. It needs to be considered that the historical strains of PHEV and PRCV used in our analysis were also of European origin. This is unlike BCoV, where the historical Mebus strain is of American origin. The effects of aa substitutions on neutralizing antibody activity are highly context-dependent, with some increasing, decreasing, or not affecting neutralization at all, depending on the specific mutation and its location within the viral protein [51]. The RBD is the principal target of neutralizing antibodies, and immune escape shown by ≥8-fold reductions in neutralization titers [52]. However, neutralizing antibodies can also target regions outside the RBD, including the N-terminal domain and fusion peptide [53]. For BCoV, there is also evidence of neutralizing antibodies targeting the HE protein, although their effect is weaker than those targeting the S protein [15,16]. The use of polyclonal swine antisera provides a broad measure of neutralization and does not allow mapping of epitope-specific responses. The neutralization activity observed reflects cumulative responses to multiple epitopes. Antigenic determinants of the PHEV RBD remain undefined. For PRCV, antigenic site A-B overlaps with the RBD, and substitutions in this domain can reduce neutralization [54,55]. Similarly, most aa mutations in the SARS-CoV-2 S protein conferring immune escape occur in the RBD [56].

Although antigenic drift was limited, the aa changes observed in S and HE proteins may still influence virus–host interactions. In other coronaviruses, aa substitutions have altered cleavage sites, tissue tropism, or increased transmissibility [2,57]. The substitutions detected in our recent isolates may therefore warrant further investigation to determine their effects on virus characteristics. Our colleagues have shown differences in replication and receptor usage in the murine neural cell line N2a between the PHEV VW572 and G/412/2020 isolate [24].

We acknowledge the limitations in this study. Isolation of these coronaviruses from field samples is challenging, as they generally do not replicate easily in cell culture [58,59,60]. This resulted in the small number of isolates obtained. Virus isolates were obtained from 25.6%, 14.5% and 57.1% of samples diagnosed to be positive by metagenomic analysis for BCoV, PHEV and PRCV, respectively. This introduces an inherent bias toward strains with higher in vitro replication competence. The results may therefore underrepresent the full genetic and antigenic diversity circulating in the field. For PHEV, the very small number of isolates (*n* = 3) and the absence of synonymous substitutions in one strain (G/412/2020) with respect to the VW572 reference strain further limit the precision of the evolutionary rate estimates, and these results should therefore be interpreted with caution. The limited number of European S and HE sequences for PHEV in the GenBank database also constrained comparative analyses.

Overall, our findings suggest that BCoV, PHEV, and PRCV remain genetically and antigenically stable within Europe. This is demonstrated by the high sequence homology and limited evidence of immune escape. However, continued surveillance is essential for BCoV, as even minor changes in surface proteins could affect the efficacy of licensed vaccines.

## Figures and Tables

**Table 1:** Overview of BCoV, PHEV and PRCV isolated annually in this study from 2020 to 2023.

Virus	Year	No. of Samples			Countries Represented by Whole Genome Sequences (*n*)
		Diagnosed by Metagenomic Analysis	Isolated in Cell Culture	Successfully Sequenced	
BCoV	2020	4	4	3	BE (3)
	2021	12	4	1	BE (1)
	2022	23	12	11	BE (10), NL (1)
	2023	7	0	0	
PHEV	2020	3	1	1	NL (1)
	2021	31	7	1	NL (1)
	2022	21	1	0	
	2023	14	3 *	1	AT (1)
PRCV	2020	1	1	1	BE (1)
	2021	11	7	7	BE (3), NL (3), DE (1)
	2022	7	3	3	BE (2), NL (1)
	2023	2	15 **	7	BE (7) ^§^

Abbreviations: BE = Belgium, NL = The Netherlands, AT = Austria, DE = Germany. * 2 pooled nasal swabs specimens obtained directly from Belgian swine farms through routine influenza surveillance. ** 14 pooled nasal swabs obtained directly from Belgian swine farms through routine influenza surveillance. ^§^ 8 pooled nasal swab samples were excluded from sequencing because they were collected on the same day from the same farms as other submitted samples.

**Table 2:** Codons identified under diversifying selection in the BCoV S and HE genes using MEME, SLAC, FEL and FUBAR.

Gene	Method	No. Sites Under Positive Selection	Codons Under Diversifying Selection	dN/dS Rate Ratio
S	MEME	57	4, 7, 12, 14, 40, 101, 112, 113, 121, 125, 146, 155, 173, 220, 260, 287, 304, 412, 441, 455, 464, 470, 499, 501, 509, 514, 528, 571, 578, 588, 592, 655, 716, 744, 769, 771, 877, 892, 893, 922, 927, 932, 1026, 1030, 1144, 1188, 1192, 1237, 1239, 1247, 1248, 1275, 1296, 1318, 1361, 1362, 1363	0.2342
	SLAC	24	9, **12**, **113**, **121**, **146**, **179**, 447, 455, 464, 465, 474, **499**, **501**, **509**, **514**, 571, 606, 716, 718, 744, **927**, 1192, **1237**, 1362	0.2337
	FEL	25	9, **12**, **113**, **121**, **146**, **179**, 447, 455, 464, 465, 474, **499**, **501**, **509**, 511, **514**, 571, 606, 716, 718, 744, **927**, 1192, **1237**, 1363	0.1852
	FUBAR	15	**12**, **113**, **121**, **146**, **179**, 441, **499**, **501**, **509**, **514**, **927**, **1237**, 1239, 1296, 1362	N/A
HE	MEME	4	65, 181, 243, 309	0.1906
	SLAC	1	**66**	0.2573
	FEL	1	**66**	0.2396
	FUBAR	4	**66**, 147, 218, 380	N/A

Codons underlined were detected by MEME + SLAC/FEL/FUBAR. Codons in bold were detected by SLAC, FEL and FUBAR methods. N/A = not applicable.

**Table 3:** Regional comparison of percent amino acid sequence identities of the S and HE surface proteins of recent BCoV, PHEV and PRCV isolates from western Europe.

Virus	Surface Protein	Sequence Type	Percent Sequence Identity of the Surface Proteins of Recent W. European Isolates of BCoV, PHEV and PRCV and … Isolates						
			Classical	African	Asian	European	North American	South American	Recent W. European
BCoV	S	nt	96.5–97.3	98.6–99.0	95.4–97.2	97.4–99.1	96.3–97.7	96.6–96.9	98.5–100
		aa	96.0–96.8	98.7–99.2	94.6–97.7	97.5–99.3	96.2–97.8	96.9–97.4	98.5–100
	HE	nt	96.4–97.6	97.8–98.8	96.0–98.1	97.9–98.8	96.6–98.0	-	97.8–100
		aa	96.0–98.1	98.4–98.8	96.0–98.8	97.9–99.3	96.7–98.8	-	98.1–100
PHEV	S	nt	96.2–100	-	94.2–98.0	97.2–98.1	95.1–96.8	-	96.9–97.2
		aa	96.9–99.9	-	94.1–98.1	96.4–97.4	94.7–96.7	-	96.4–96.8
	HE	nt	95.4–99.8	-	92.8–100	-	94.0–96.2	-	96.9–99.6
		aa	93.4–99.3	-	91.3–100	-	92.0–95.8	-	96.2–99.5
PRCV	S	nt	96.3–96.9	-	92.5–96.6	96.4–97.6	92.4–94.9	-	98.5–100
		aa	96.3–96.9	-	94.7–96.5	96.5–97.8	93.2–95.5	-	98.6–100

nt = nucleotide; aa = amino acid, - = no data.

**Table 4:** Codons identified under diversifying selection in the PHEV S and HE genes using MEME, SLAC, FEL and FUBAR.

Gene	Method	No. Sites Under Positive Selection	Codon Under Positive Selection	dN/dS Rate Ratio
S	MEME	16	5, 11, 25, 63, 113, 154, 169, 479, 568, 696, 770, 1012, 1167, 1244, 1261, 1330	0.2415
	SLAC	9	**11**, **25**, **154**, **770**, **842**, **1012**, 1244, **1330**, 1349	0.2544
	FEL	13	**11**, **25**, **154**, 352, 696, 769, **770**, **842**, **1012**, 1034, 1198, 1244, **1330**	0.2415
	FUBAR	18	**11**, **25**, **154**, 557, 568, **770**, **842**, 959, **1012**, 1034, 1167, 1198, 1235, 1261, 1264, **1330**, 1339, 1342	N/A
HE	MEME	5	2, 160, 211, 365, 411	0.2311
	SLAC	0	-	0.2866
	FEL	3	24, 160, 411	0.2814
	FUBAR	1	411	N/A

Codons underlined were detected by MEME + SLAC/FEL/FUBAR. Codons in bold were detected by SLAC, FEL and FUBAR methods. N/A = not applicable.

**Table 5:** Codons identified under diversifying selection in the PRCV S gene using MEME, SLAC and FEL with *p*-value of <0.1.

Gene	Method	No. Sites Under Positive Selection	Codon Under Positive Selection	dN/dS Rate Ratio
S	MEME	15	3, 5, 22, 23, 41, 180, 204, 214, 355, 360, 366, 374, 619, 621, 753	0.2091
	SLAC	0	-	0.2339
	FEL	1	180	0.2091
	FUBAR	4	22, 396, 621, 753	N/A

Codons underlined were detected by MEME + SLAC/FEL/FUBAR. N/A = not applicable.

## Data Availability

The whole genome sequences generated in this study have all been deposited in NCBI’s GenBank database.

## Supplementary Materials

The following supporting information can be downloaded at: https://www.mdpi.com/article/10.3390/v18070705/s1, Table S1: List of BCoV, PHEV and PRCV isolates obtained in this study, their origin and year of isolation; Table S2: Gene sequences selected from GenBank for determination of the evolutionary rates of the S and HE surface proteins of BCoV, PHEV and PRCV; Table S3: dN/dS ratios for the S and HE genes of BCoV, PHEV and PRCV isolated in Europe between 2020 and 2023 with reference to historic virus strains; Figure S1: Pairwise amino acid sequence Identities of the S and HE surface proteins of BCoV, PHEV and PRCV.

## Abbreviations

The following abbreviations are used in this manuscript:

aa	Amino acid
BCoV	Bovine coronavirus
HE	Hemagglutinin esterase
nt	nucleotide
PHEV	Porcine hemagglutinin encephalomyelitis virus
PRCV	Porcine respiratory coronavirus
S	Spike
RBD	Receptor binding domain
VN	Virus neutralization
