# Correction: Mumo et al. Genetic and Antigenic Characterization of Bovine and Porcine Respiratory Coronaviruses Circulating in Western Europe, 2020–2023. *Viruses* 2026, *18*, 705

**DOI:** 10.3390/v18080837

**Published:** 2026-07-30

**Authors:** Ruth M. Mumo, Anna Parys, Sieglinde Coppens, Nick Vereecke, Sebastiaan Theuns, Bart Pardon, Kristien Van Reeth

**Affiliations:** 1 Laboratory of Virology, Department of Translational Physiology, Infectiology and Public Health, Faculty of Veterinary Medicine, Ghent University, Salisburylaan 133, 9820 Merelbeke, Belgium; ruth3mumo@gmail.com (R.M.M.); nickvereecke@hotmail.com (N.V.); 2 National Reference Centre for Respiratory Pathogens, Service of Viral Diseases, Sciensano, Engelandstraat 642, 1050 Brussels, Belgium; anna.parys@sciensano.be; 3 PathoSense BV, Poortakkerstraat 41A, 9051 Gent, Belgium; sieglinde.coppens@pathosense.com (S.C.); sebastiaan.theuns@pathosense.com (S.T.); 4 Calf Health Research Group, Clinic for Ruminants, Department of Internal Medicine, Reproduction and Population Medicine, Faculty of Veterinary Medicine, Ghent University, Salisburylaan 133, 9820 Merelbeke, Belgium; bart.pardon@ugent.be

**Addition of Authors**

Anna Parys and Nick Vereecke were not included as authors in the original publication [1]. The order of authors was modified, with Anna Parys and Nick Vereecke placed in the second and fourth positions, respectively. It was agreed by all authors that Anna Parys and Nick Vereecke contributed significantly to the work. The corrected author contributions statement appears here: Conceptualization, R.M.M., A.P. and K.V.R.; methodology, R.M.M., A.P., N.V., S.T. and K.V.R.; formal analysis, R.M.M., N.V. and S.C.; investigation, R.M.M., A.P. and K.V.R.; resources, S.T., B.P. and K.V.R.; data curation, R.M.M.; writing—original draft preparation, R.M.M. and K.V.R.; writing—review and editing, R.M.M., A.P., S.C., N.V., S.T., B.P. and K.V.R.; visualization, R.M.M.; project administration, K.V.R.; funding acquisition, K.V.R. All authors have read and agreed to the published version of the manuscript.

**Missing Funding**

The funding information was adjusted to include that Nick Vereecke was funded by the Flemish Agency for Innovation and Entrepreneurship (Baekeland Mandate HBC.2020.2889).

**Addition to Conflicts of Interest Statement**

This section was updated to include that Nick Vereecke is a former employee of PathoSense.

The authors state that the scientific conclusions are unaffected. These corrections were approved by the Academic Editor. The original publication has also been updated. All authors have read and agreed to the published version of the manuscript.
