# Supplementary material for: Genetic and Antigenic Characterization of Bovine and Porcine Respiratory Coronaviruses Circulating in Western Europe, 2020–2023
Source: Viruses. 2026 Jun 26;18(7):705. doi: 10.3390/v18070705 (PMC13431604; doi:10.3390/v18070705)
Supplement: Supplementary file 1 [file viruses-18-00705-s001.zip › Supplementary material.pdf]

Table S1: List of BCoV, PHEV and PRCV isolates obtained in this study, their origin and year of isolation

Isolate name	Abbreviation	Origin	Year isolated	Accession No.
BCoV-Gent/PS-639/2020	G/639/2020	Belgium	2020	PX418135
BCoV-Gent/PS-640/2020	G/640/2020	Belgium	2020	PX418136
BCoV-Gent/PS-666/2020	G/666/2020	Belgium	2020	PX447284
BCoV-Gent/PS-3199/2021	G/3199/2021	Belgium	2021	PX447285
BCoV-Gent/PS-3202/2022	G/3202/2022	Belgium	2022	PX418137
BCoV-Gent/PS-3662/2022	G/3662/2022	Belgium	2022	PX580609
BCoV-Gent/PS-3667/2022	G/3667/2022	Belgium	2022	PX580610
BCoV-Gent/PS-4367/2022	G/4367/2022	Belgium	2022	PX418138
BCoV-Gent/PS-4381/2022	G/4381/2022	Belgium	2022	PX447286
BCoV-Gent/PS-4382/2022	G/4382/2022	Belgium	2022	PX418139
BCoV-Gent/PS-4415/2022	G/4415/2022	Belgium	2022	PX418140
BCoV-Gent/PS-4416/2022	G/4416/2022	Belgium	2022	PX418141
BCoV-Gent/PS-4418/2022	G/4418/2022	Belgium	2022	PX418142
BCoV-Gent/PS-4419/2022	G/4419/2022	Belgium	2022	PX447287
BCoV-Gent/PS-4420/2022	G/4420/2022	Belgium	2022	PX418143
PHEV-Gent/PS-412/2020	G/412/2020	The Netherlands	2020	PV820712
PHEV-Gent/PS-766/2021	G/766/2021	The Netherlands	2021	PX580612
PHEV-Gent/PS-7664/2023	G/7664/2023	Austria	2023	PX447288
PRCV isolate TLM83	TLM83	Belgium	1984	PX580611
PRCV isolate 89V367	89V367	Belgium	1989	PX680842
PRCV isolate 89V431	89V431	Belgium	1989	PX418144
PRCV isolate 90V170	90V170	Belgium	1990	PX418145
PRCV-Gent/PS-071/2020	G/071/2020	Belgium	2020	PX418146
PRCV-Gent/PS-721/2021	G/721/2021	Belgium	2021	PX418147
PRCV-Gent/PS-794/2021	G/794/2021	The Netherlands	2021	PX418148
PRCV-Gent/PS-803/2021	G/803/2021	The Netherlands	2021	PX418149
PRCV-Gent/PS-811/2021	G/811/2021	The Netherlands	2021	PX418150
PRCV-Gent/PS-1146/2021	G/1146/2021	Belgium	2021	PX418151
PRCV-Gent/PS-1538/2021	G/1538/2021	Germany	2021	PX418152
PRCV-Gent/PS-2061/2021	G/2061/2021	Belgium	2021	PX418153
PRCV-Gent/PS-2269/2022	G/2269/2022	Belgium	2022	PX418154
PRCV-Gent/PS-2271/2022	G/2271/2022	Belgium	2022	PX418155
PRCV-Gent/PS-3063/2022	G/3063/2022	Belgium	2022	PX418156
PRCV-Gent/PS-2196/2023	G/2196/2023	Belgium	2023	PX418157
PRCV-Gent/85-3/2023	G/85-3/2023	Belgium	2023	PX418158
PRCV-Gent/86-1/2023	G/86-1/2023	Belgium	2023	PX418159
PRCV-Gent/87-4/2023	G/87-4/2023	Belgium	2023	PX418160
PRCV-Gent/90-6/2023	G/90-6/2023	Belgium	2023	PX418163
PRCV-Gent/91-1/2023	G/91-1/2023	Belgium	2023	PX418161
PRCV-Gent/95-1/2023	G/95-1/2023	Belgium	2023	PX418162

Table S2: Gene sequences selected from GenBank for determination of the evolutionary rates of the S and HE surface proteins of BCoV, PHEV and PRCV

Table S2A: BCoV S gene sequences from GenBank included in the evolutionary rate analysis

Accession No	Origin	Year isolated	Accession No	Origin	Year isolated	Accession No	Origin	Year isolated
AF220295.1	Canada	1999	PQ313104.1	China	2023	LC494140.1	Japan	2015
KM985631.1	China	2014	PP101630.1	China	2023	LC494146.1	Japan	2016
MK095175.1	China	2018	OR454204.1	China	2023	LC494152.1	Japan	2017
MK095176.1	China	2017	OR612022.1	China	2022	LC494155.1	Japan	2014
MK095178.1	China	2018	OR621176.1	China	2022	LC494156.1	Japan	2014
MK095179.1	China	2018	OR596700.1	China	2022	LC494158.1	Japan	2016
MK095181.1	China	2018	OR603989.1	China	2022	LC494159.1	Japan	2017
MK095183.1	China	2018	OR077312.1	China	2023	LC494168.1	Japan	2009
MK095184.1	China	2018	OR077315.1	China	2022	LC494171.1	Japan	2010
MK095186.1	China	2018	OR088593.1	China	2023	LC494172.1	Japan	2007
MN982174.1	China	2018	OP866727.1	China	2021	LC494173.1	Japan	2008
MN982181.1	China	2018	OP866728.1	China	2021	LC494175.1	Japan	2016
MW711290.1	China	2019	ON142315.1	China	2021	LC494177.1	Japan	2008
MW711291.1	China	2019	ON142316.1	China	2021	LC494184.1	Japan	2017
MW711292.1	China	2019	ON142320.1	China	2021	AB354579.1	Japan	1976
MW711294.1	China	2020	ON093194.1	China	2021	PV345614.1	Kazakhstan	2024
MW711297.1	China	2020	MN982198.1	China	2018	AY935646.1	S. Korea	2003
MW711298.1	China	2020	MN982199.1	China	2018	DQ389634.1	S. Korea	2004
MW711303.1	China	2020	KU886219.1	China	2015	DQ389635.1	S. Korea	2004
MZ603735.1	China	2021	PP273504.1	China	2019	DQ389636.1	S. Korea	2004
MT975570.1	China	2019	PP599028.1	China	2023	DQ389652.1	S. Korea	2003
MT975571.1	China	2018	OR753439.1	China	2023	DQ389653.1	S. Korea	2003
MW521175.1	China	2020	HE616739.1	Cuba	2010	DQ389654.1	S. Korea	2003
MW521177.1	China	2020	KF169913.1	Denmark	2003	DQ389655.1	S. Korea	2003
MW521179.1	China	2020	KF169914.1	Denmark	2003	DQ389656.1	S. Korea	2003
MW521184.1	China	2020	KF169915.1	Denmark	2003	DQ389657.1	S. Korea	2003
MW521185.1	China	2020	KF169917.1	Denmark	2005	DQ389658.1	S. Korea	2003
MW521187.1	China	2020	PV061390.1	Ethiopia	2023	DQ389659.1	S. Korea	2003
MW521188.1	China	2020	PV061391.1	Ethiopia	2023	DQ389660.1	S. Korea	2003
MW521191.1	China	2020	KT318112.1	France	2005	HM573326.1	S. Korea	2010
MW521193.1	China	2020	KT318114.1	France	2008	OP186313.1	S. Korea	2017
MW521194.1	China	2020	KT318115.1	France	2003	OP186320.1	S. Korea	2020
MZ711344.1	China	2020	KT318116.1	France	2010	OP186324.1	S. Korea	2018
MZ711348.1	China	2020	KT318121.1	France	2013	OR464033.1	S. Korea	2022
MZ711349.1	China	2020	KT318122.1	France	2014	OR464036.1	S. Korea	2022
MZ711352.1	China	2020	MG757143.1	France	2014	OR464037.1	S. Korea	2022
MZ711358.1	China	2021	MG757144.1	France	2014	OR464041.1	S. Korea	2022
MZ711360.1	China	2021	MG757138.1	France	2014	OR464043.1	S. Korea	2022
OR124732.1	China	2022	KX982264.1	France	2014	KF169908.1	Sweden	1992
OR136878.1	China	2021	KF272919.1	Ireland	2011	KF169910.1	Sweden	2002
OR947445.1	China	2022	OR271252.1	Ireland	2022	KF169921.1	Sweden	2005
OR947446.1	China	2021	ON792944.1	Ireland	2019	KF169922.1	Sweden	2006
OR947447.1	China	2022	ON792964.1	Ireland	2020	KF169923.1	Sweden	2006
OR947451.1	China	2022	EU814647.1	Italy	2006	KF169924.1	Sweden	2006
OR947455.1	China	2022	LC830477.1	Japan	2022	KF169932.1	Sweden	2008
PQ110069.1	China	2023	LC642814.1	Japan	2020	KF169933.1	Sweden	2008
PQ243293.1	China	2024	LC494126.1	Japan	2016	KF169934.1	Sweden	2009
OP296992.1	Taiwan	2017	LC494129.1	Japan	2010	KF169935.1	Sweden	2009
OR502440.1	USA	2021	OP037406.1	USA	2021	OP037440.1	USA	2022
OR502441.1	USA	2021	OP037407.1	USA	2021	OP037442.1	USA	2022
OP037367.1	USA	2014	OP037409.1	USA	2021	MH043952.1	USA	2017
OP037371.1	USA	2015	OP037412.1	USA	2022	MH043953.1	USA	2017
OP037372.1	USA	2015	OP037417.1	USA	2018	FJ938066.1	USA	1996
OP037385.1	USA	2017	OP037418.1	USA	2018	EF424615.1	USA	2001
OP037389.1	USA	2019	OP037420.1	USA	2018	EF424617.1	USA	2001
OP037390.1	USA	2019	OP037421.1	USA	2018	EF424620.1	USA	2000
OP037393.1	USA	2020	OP037427.1	USA	2018	DQ811784.2	USA	1983
OP037394.1	USA	2021	OP037428.1	USA	2018	AF391542.1	USA	2001
OP037396.1	USA	2021	OP037430.1	USA	2019	AF391541.1	USA	2001
OP037397.1	USA	2020	OP037431.1	USA	2019	U00735.2	USA	1972
OP037398.1	USA	2020	OP037432.1	USA	2019	MH203064.1	Vietnam	2017
OP037399.1	USA	2020	OP037434.1	USA	2019	MK046003.1	Vietnam	2017
OP037400.1	USA	2020	OP037435.1	USA	2019	MK046004.1	Vietnam	2018
OP037403.1	USA	2020	OP037436.1	USA	2022	MK046008.1	Vietnam	2018
OP037404.1	USA	2021	OP037437.1	USA	2022	MK046011.1	Vietnam	2018
OP037405.1	USA	2021	OP037439.1	USA	2022			

Accession No	Origin	Year isolated	Accession No	Origin	Year isolated	Accession No	Origin	Year isolated
OP037408.1	USA	2021	ON146444.1	USA	2014			

Table S2B: BCoV HE gene sequences from GenBank included in the evolutionary rate analysis

Accession No	Origin	Year isolated	Accession No	Origin	Year isolated	Accession No	Origin	Year isolated
MK095137.1	China	2017	OR621174.1	China	2022	OP037442.1	USA	2022
MK095142.1	China	2018	OR621176.1	China	2022	ON792959.1	Ireland	2019
MK095144.1	China	2018	OQ161705.1	Turkey	2019	LC494132.1	Japan	2011
MN982193.1	China	2018	OR077313.1	China	2022	LC494140.1	Japan	2015
OP762501.1	China	2021	OR077316.1	China	2022	LC494173.1	Japan	2008
PP273504.1	China	2019	OP296992.1	Taiwan	2017	LC494177.1	Japan	2008
PP599028.1	China	2023	OP037384.1	USA	2017	MG757141.1	France	2014
PV061390.1	Ethiopia	2023	OP037393.1	USA	2020	MH043952.1	USA	2017
PV061391.1	Ethiopia	2023	OP037400.1	USA	2020	KU886219.1	China	2015
PQ588962.1	China	2024	OP037403.1	USA	2020	AB354579.1	Japan	1976
PQ313104.1	China	2023	OP037404.1	USA	2021	EF424615.1	USA	2001
LC830477.1	Japan	2022	OP037412.1	USA	2022	DQ811784.2	USA	1983
OR502441.1	USA	2021	OP037418.1	USA	2018	U00735.2	USA	1972
OR502442.1	USA	2021	OP037427.1	USA	2018	AF220295.1	Canada	1999
PP101630.1	China	2023	OP037430.1	USA	2019	OP037442.1	USA	2022
OR753439.1	China	2023	OP037435.1	USA	2019			
OR454204.1	China	2023	OP037436.1	USA	2022			

Table S2C: PHEV S gene sequences from GenBank included in the evolutionary rate analysis

Accession No	Origin	Year isolated	Accession No	Origin	Year isolated	Accession No	Origin	Year isolated
KY492680.1	The Netherlands	2015	KY419105.1	USA	2015	PP646323.1	China	2023
OQ798832.1	China	2020	KY419113.1	USA	2015	PP646324.1	China	2023
OQ798833.1	China	2020	DQ011855.1	Belgium	1972	PP646325.1	China	2023
PP646298.1	China	2021	PP646301.1	China	2021	PP646326.1	China	2023
PP646299.1	China	2021	PP646302.1	China	2021	PP646327.1	China	2023
PV730382.1	China	2025	PP646303.3	China	2022	PP646328.1	China	2023
PQ241464.1	USA	2021	PP646304.1	China	2022	PP646329.1	China	2023
OQ798806.1	China	2018	PP646305.1	China	2022	PP646330.1	China	2023
OQ798807.1	China	2021	PP646306.1	China	2022	PP646331.1	China	2023
OQ798808.1	China	2021	PP646307.1	China	2022	PP646332.1	China	2023
OQ798809.1	China	2021	PP646308.1	China	2022	PP646333.1	China	2023
OQ798810.1	China	2019	PP646309.1	China	2022	PP646334.1	China	2023
OQ798818.1	China	2021	PP646311.1	China	2022	PP646335.1	China	2023
OQ798819.1	China	2021	PP646312.1	China	2022	PP646336.1	China	2023
OQ798821.1	China	2021	PP646313.1	China	2022	PP646337.1	China	2023
OQ798825.1	China	2016	PP646314.1	China	2022	PP646338.1	China	2023
OQ7988826.1	China	2020	PP646315.1	China	2022	PP646340.1	China	2023
OQ305205.1	China	2017	PP646316.1	China	2022	PP646341.1	China	2023
OQ305206.1	China	2017	PP646317.1	China	2023	PP646342.1	China	2023
OQ305207.1	China	2017	PP646318.1	China	2023	PP646343.1	China	2023
OL542832.1	S. Korea	2021	PP646319.1	China	2023	PP646344.1	China	2023
MW165134.1	USA	1970	PP646320.1	China	2023	PP646345.1	China	2023
KY994645.1	China	2008	PP646321.1	China	2023	PP646347.1	China	2024
KY419103.1	USA	2015	PP646322.1	China	2023			

Table S2D: PHEV HE gene sequences from GenBank included in the evolutionary rate analysis

Accession No	Origin	Year isolated	Accession No	Origin	Year isolated	Accession No	Origin	Year isolated
MW165134.1	USA	1970	OQ798806.1	China	2018	OQ798814.1	China	2021
DQ011855.1	Belgium	1972	OQ798810.1	China	2019	OQ798816.1	China	2021
KY994645.1	China	2008	OQ798811.1	China	2019	OQ798818.1	China	2021
MF083115.1	China	2014	OQ798827.1	China	2019	OQ798819.1	China	2021
KY419103.1	USA	2015	OQ798829.1	China	2019	OQ798820.1	China	2021
KY419110.1	USA	2015	OQ798812.1	China	2020	OQ798821.1	China	2021
KY419112.1	USA	2015	OQ798817.1	China	2020	OQ798822.1	China	2021
KY419113.1	USA	2015	OQ798826.1	China	2020	OQ798823.1	China	2021
KY419107.1	USA	2015	OQ798828.1	China	2020	OL542832.1	S. Korea	2021
KY419111.1	USA	2015	PQ241464.1	USA	2021	OP959790.1	China	2021

OQ798825.1	China	2016	OQ798807.1	China	2021	PV730382.1	China	2025
OQ305205.1	China	2017	OQ798808.1	China	2021	OQ798814.1	China	2021
OQ305208.1	China	2017	OQ798809.1	China	2021	OQ798816.1	China	2021

Table S2E: PRCV S gene sequences from GenBank included in the evolutionary rate analysis

Accession No	Origin	Year isolated	Accession No	Origin	Year isolated	Accession No	Origin	Year isolated
OM830818.1	UK	1986	KR270796.1	USA	2014	PQ204801.1	China	2023
M94097.1	The Netherlands	1987	KY406735.1	USA	2016	PQ204809.1	China	2024
OK078898.1	Denmark	1990	OR209254.1	USA	2020	PQ204810.1	China	2024
OR689864.1	Belgium	1991	PQ204807.1	China	2023	PV096984.1	China	2024
OR689863.1	Italy	2012	PQ204808.1	China	2023	PP781502.1	S. Korea	2024
OM830321.1	USA	1990	PQ204804.1	China	2023	PP781503.1	S. Korea	2024
OR209252.1	USA	1991	PQ204805.1	China	2023			
OR209253.1	USA	1992	PQ204800.1	China	2023			

Table S3: dN/dS ratios for the S and HE genes of BCoV, PHEV and PRCV isolated in Europe between 2020 and 2023 with reference to historic virus strains.

BCoV isolates	S gene	HE gene	PHEV isolates	S gene	HE gene	PRCV isolates	S gene
Mebus/1972	Ref	Ref	VW572/1972	Ref	Ref	91V44/1991	Ref
G/639/2020	0.22	0.12	G/412/2020	Undefined	Undefined	G/071/2020	0.16
G/640/2020	0.19	0.10	G/766/2021	0.16	0.30	G/721/2021	0.15
G/666/2020	0.20	0.12	G/7664/2023	0.25	0.26	G/794/2021	0.16
G/3199/2021	0.23	0.20				G/803/2021	0.15
G/3202/2022	0.21	0.12				G/811/2021	0.17
G/3662/2022	0.23	0.17				G/1146/2021	0.15
G/3667/2022	0.23	0.17				G/1538/2021	0.14
G/4367/2022	0.22	0.12				G/2061/2021	0.16
G/4381/2022	0.22	0.10				G/2269/2022	0.15
G/4382/2022	0.22	0.19				G/2271/2022	0.15
G/4415/2022	0.22	0.12				G/3063/2022	0.16
G/4416/2022	0.22	0.13				G/2196/2023	0.15
G/4418/2022	0.22	0.12				G/85-3/2023	0.15
G/4419/2022	0.22	0.12				G/86-1/2023	0.15
G/4420/2022	0.22	0.12				G/87-4/2023	0.14
						G/90-6/2023	0.14
						G/91-1/2023	0.15
						G/95-1/2023	0.16

Ref = reference strain

Figure S1: Pairwise amino acid sequence Identities of the S and HE surface proteins of BCoV, PHEV and PRCV

#	BCoV strains/isolates	1	2	3	4	5	6	7	8	9	10	11	12	13	14	15	16	17	18	19	20	21	22	23	24	25	26	27	28	29	30	31	32	33	34	35	36	37	38	39	40	41
1	**U00735.2 USA_1972**		99.0	97.8	99.3	99.6	96.1	97.4	96.9	96.9	97.8	97.1	98.6	96.3	95.8	97.3	96.7	96.9	96.7	96.4	96.1	96.3	96.2	96.6	96.4	96.1	96.2	96.3	96.3	96.3	96.3	96.1	96.3	96.3	96.3	96.3	96.3	97.8	96.0	96.9	96.9	98.5
2	AB354579.1 Japan_1976	99.6		98.2	99.4	99.1	96.4	97.9	97.3	97.2	97.1	97.4	99.3	96.6	96.0	97.7	97.1	97.3	97.1	96.8	96.5	96.6	96.6	96.8	96.8	96.4	96.4	96.7	96.7	96.5	96.7	96.5	96.6	96.6	96.6	96.6	96.6	98.2	96.3	97.3	97.1	98.8
3	D00731.1 France_1979	98.3	98.4		98.0	97.7	96.3	97.7	97.2	97.1	96.1	97.4	97.5	96.6	96.1	97.6	97.0	97.2	97.0	96.7	96.6	96.5	96.5	96.7	96.7	96.2	96.3	96.6	96.6	96.6	96.6	96.5	96.6	96.6	96.6	96.6	96.6	98.0	96.3	97.1	97.1	97.5
4	EF193075.1 Germany_1983	99.7	99.8	98.3		99.3	96.3	97.7	97.1	97.1	97.2	97.3	99.0	96.4	95.8	97.5	96.9	97.1	96.9	96.6	96.3	96.4	96.4	96.6	96.6	96.3	96.3	96.6	96.6	96.3	96.6	96.3	96.5	96.5	96.5	96.5	96.5	98.0	96.2	97.1	97.0	98.9
5	AF220295.1 Canada_1999	99.8	99.7	98.2	99.7		96.0	97.4	96.9	96.8	97.6	97.0	98.7	96.4	95.5	97.2	96.6	96.9	96.6	96.3	96.1	96.3	96.1	96.6	96.3	96.0	96.1	96.3	96.3	96.2	96.3	96.1	96.2	96.2	96.2	96.2	96.2	97.8	96.1	96.9	96.9	98.7
6	PV061390.1 Ethiopia_2023	96.9	97.1	96.6	97.0	96.9		97.3	97.2	97.4	94.6	97.5	95.8	96.6	95.9	97.7	97.8	98.8	99.0	98.5	98.8	98.9	99.2	99.1	99.2	98.7	98.8	99.2	99.2	99.0	99.2	98.9	99.0	99.0	99.0	99.0	99.0	97.4	96.5	97.4	97.3	95.9
7	AY935646.1 South_Korea_2003	98.1	98.3	97.9	98.2	98.1	97.0		98.3	98.2	96.0	98.5	97.3	97.4	96.6	98.0	97.7	98.1	97.9	97.6	97.4	97.4	97.4	97.7	97.7	97.1	97.3	97.6	97.6	97.4	97.6	97.5	97.5	97.5	97.5	97.5	97.5	98.5	97.3	98.5	98.2	97.5
8	LC494126.1 Japan_2016	97.4	97.5	97.1	97.4	97.3	96.7	98.3		98.9	95.5	98.9	96.6	98.2	97.2	98.1	98.0	98.0	97.7	97.5	97.1	97.5	97.2	97.4	97.4	96.9	97.2	97.5	97.5	97.1	97.4	97.1	97.3	97.3	97.3	97.3	97.3	98.2	98.1	98.2	98.9	96.8
9	MK095176.1 China_2017	97.4	97.6	97.2	97.6	97.4	96.6	98.6	98.5		95.5	99.0	96.6	98.5	97.4	98.0	97.9	98.0	97.8	97.4	97.3	97.5	97.4	97.5	97.6	97.1	97.2	97.5	97.5	97.3	97.6	97.3	97.4	97.4	97.4	97.4	97.4	98.1	98.2	98.5	99.0	96.7
10	OP296992.1 Taiwan_2017	99.2	98.9	97.6	98.9	99.1	96.3	97.6	96.9	96.9		95.7	96.6	95.0	94.4	95.7	95.2	95.5	95.2	95.0	94.7	95.0	94.7	95.2	95.0	94.6	94.7	94.9	94.9	94.8	95.0	94.7	94.8	94.8	94.8	94.8	94.8	96.4	94.6	95.5	95.5	96.7
11	MK046011.1 Vietnam_2018	97.4	97.6	97.1	97.6	97.4	96.7	98.4	98.3	98.8	96.9		96.8	98.2	97.2	98.0	98.0	98.2	98.0	97.8	97.4	97.7	97.5	97.7	97.7	97.2	97.3	97.7	97.7	97.4	97.7	97.4	97.6	97.6	97.6	97.6	97.6	98.4	98.2	98.8	99.1	96.9
12	OR464041.1 South_Korea_2022	99.4	99.7	98.1	99.6	99.5	96.8	98.1	97.2	97.3	98.7	97.3		95.9	95.3	97.0	96.4	96.6	96.4	96.1	95.9	95.9	95.9	96.1	96.1	95.8	95.8	96.1	96.1	95.8	96.1	95.8	96.0	96.0	96.0	96.0	96.0	97.5	95.7	96.6	96.5	98.6
13	PQ243294.1 China_2024	97.2	97.3	97.0	97.2	97.2	96.4	98.0	98.1	98.6	96.7	98.0	97.1		96.9	97.2	97.1	97.2	97.1	97.0	96.4	96.9	96.6	97.0	96.8	96.3	96.9	96.7	96.7	96.8	96.8	96.5	96.8	96.8	96.8	96.8	96.8	97.3	98.2	97.8	98.2	96.3
14	PV345614.1 Kazakhstan_2024	96.1	96.2	95.8	96.2	96.0	95.5	96.8	96.9	97.4	95.6	97.3	96.0	96.7		96.6	96.3	96.5	96.3	95.8	95.8	96.1	95.8	96.1	96.1	95.5	95.9	96.0	96.0	95.9	96.1	95.8	96.0	96.0	96.0	96.0	96.0	96.7	96.6	96.6	97.3	95.3
15	KF169908.1 Sweden_1992	98.1	98.3	97.8	98.2	98.1	97.5	98.1	97.5	97.6	97.5	97.5	98.0	97.2	96.2		98.5	98.4	98.1	97.8	97.7	97.9	97.8	98.0	98.0	97.5	97.7	98.0	98.0	97.8	98.0	97.7	97.9	97.9	97.9	97.9	97.9	98.4	97.1	97.7	97.8	97.1
16	KF169913.1 Denmark_2003	97.7	97.9	97.5	97.8	97.7	97.8	97.8	97.4	97.5	97.2	97.4	97.6	97.2	96.2	98.5		98.8	98.5	98.0	97.9	98.0	98.0	98.2	98.2	97.7	97.9	98.2	98.2	98.0	98.2	97.9	98.0	98.0	98.0	98.0	98.0	98.0	97.1	97.7	97.8	96.6
17	EU814647.1 Italy_2006	97.8	98.0	97.5	97.9	97.8	98.5	97.9	97.4	97.5	97.3	97.4	97.7	97.1	96.2	98.2	98.7		99.3	99.0	98.6	98.7	98.8	99.0	99.0	98.5	98.7	99.1	99.1	98.8	99.0	98.7	98.8	98.8	98.8	98.8	98.8	98.2	97.2	98.0	98.0	96.8
18	KT318112.1 France_2005	97.6	97.8	97.4	97.7	97.6	98.6	97.7	97.3	97.3	97.1	97.3	97.5	97.0	96.1	98.1	98.6	99.1		99.0	98.8	99.0	99.1	99.3	99.3	98.8	99.0	99.3	99.3	99.1	99.3	99.0	99.1	99.1	99.1	99.1	99.1	98.0	97.0	97.9	97.7	96.6
19	KF169940.1 Sweden_2010	97.4	97.5	97.2	97.4	97.3	98.3	97.6	97.2	97.2	96.8	97.1	97.2	97.0	95.8	97.9	98.2	99.0	98.9		98.2	98.3	98.5	98.7	98.7	98.2	98.5	98.8	98.8	98.5	98.6	98.3	98.5	98.5	98.5	98.5	98.5	97.7	97.1	97.7	97.5	96.3
20	MG757143.1 France_2014	97.2	97.4	97.0	97.3	97.2	98.8	97.4	97.0	97.0	96.7	97.0	97.1	96.6	95.8	97.9	98.1	98.7	98.6	98.5		98.6	98.9	98.9	98.8	98.3	98.7	98.9	98.9	99.0	98.9	98.7	98.8	98.8	98.8	98.8	98.8	97.5	96.4	97.3	97.3	96.1
21	OR271252.1 Ireland_2022	97.2	97.3	96.9	97.2	97.2	98.7	97.2	97.0	96.9	96.7	96.9	97.1	96.7	95.8	97.8	98.0	98.6	98.6	98.4	98.9		98.9	99.2	99.0	98.5	98.8	99.3	99.3	99.0	99.0	98.8	98.9	98.9	98.9	98.9	98.9	97.6	96.6	97.5	97.4	96.1
22	G/639/2020 Belgium_2020	97.0	97.1	96.7	97.0	96.9	99.0	97.1	96.7	96.7	96.4	96.6	96.8	96.4	95.5	97.6	97.8	98.5	98.6	98.3	98.9	98.6		99.3	99.3	98.8	98.9	99.3	99.3	99.2	99.3	99.2	99.3	99.3	99.3	99.3	99.3	97.6	96.5	97.4	97.4	96.1
23	G/640/2020 Belgium_2020	97.1	97.1	96.7	97.1	97.1	98.7	97.1	96.8	96.8	96.5	96.7	96.9	96.7	95.6	97.6	97.9	98.5	98.5	98.3	98.9	98.8	99.0		99.3	98.8	99.5	99.5	99.5	99.6	99.5	99.1	99.3	99.3	99.3	99.3	99.3	97.7	96.6	97.5	97.4	96.3
24	G/666/2020 Belgium_2020	97.0	97.1	96.8	97.0	96.9	98.9	97.1	96.7	96.7	96.4	96.7	96.8	96.4	95.5	97.7	97.9	98.6	98.8	98.4	98.8	98.6	99.0	98.7		99.5	99.0	99.4	99.4	99.1	99.4	99.1	99.3	99.3	99.3	99.3	99.3	97.8	96.7	97.6	97.4	96.3
25	G/3199/2021 Belgium_2021	97.0	97.1	96.8	97.0	96.9	98.9	97.0	96.6	96.6	96.4	96.6	96.8	96.3	95.4	97.6	97.9	98.5	98.7	98.4	98.7	98.6	99.0	98.6	99.7		98.5	98.9	98.9	98.6	98.9	98.6	98.8	98.8	98.8	98.8	98.8	97.4	96.2	97.2	96.9	95.9
26	G/3202/2022 The Netherlands_2022	96.8	97.0	96.5	96.9	96.8	98.6	97.0	96.6	96.6	96.3	96.5	96.7	96.4	95.5	97.4	97.7	98.3	98.3	98.2	98.8	98.7	98.8	99.4	98.5	98.5		99.1	99.1	99.3	99.1	98.8	98.9	98.9	98.9	98.9	98.9	97.4	96.5	97.1	97.1	95.9
27	G/3662/2022 Belgium_2022	97.2	97.3	96.9	97.2	97.1	99.0	97.2	97.0	96.8	96.6	96.9	97.1	96.6	95.7	97.8	98.0	98.6	98.7	98.4	99.0	99.0	99.1	98.9	98.9	98.9	98.8		100	99.3	99.6	99.1	99.3	99.3	99.3	99.3	99.3	97.7	96.6	97.5	97.4	96.2
28	G/3667/2022 Belgium_2022	97.2	97.3	96.9	97.2	97.1	99.0	97.2	97.0	96.8	96.6	96.9	97.1	96.6	95.7	97.8	98.0	98.6	98.7	98.4	99.0	99.0	99.1	98.9	98.9	98.9	98.8	100		99.3	99.6	99.1	99.3	99.3	99.3	99.3	99.3	97.7	96.6	97.5	97.4	96.2
29	G/4367/2022 Belgium_2022	97.0	97.1	96.8	97.1	97.0	98.7	97.1	96.8	96.8	96.5	96.7	96.9	96.6	95.6	97.6	97.9	98.5	98.5	98.3	99.0	98.8	99.0	99.8	98.7	98.6	99.4	98.9	98.9		99.3	98.9	99.0	99.0	99.0	99.0	99.0	97.5	96.4	97.3	97.3	96.0
30	G/4381/2022 Belgium_2022	97.0	97.1	96.8	97.1	97.0	98.8	97.1	96.7	96.7	96.4	96.7	96.9	96.4	95.5	97.6	97.9	98.5	98.4	98.3	99.0	98.7	99.2	98.9	98.8	98.7	98.9	98.9	98.9	98.9		99.1	99.3	99.3	99.3	99.3	99.3	97.7	96.7	97.5	97.4	96.2
31	G/4382/2022 Belgium_2022	97.1	97.3	96.9	97.2	97.1	99.0	97.2	96.8	96.8	96.6	96.7	97.0	96.5	95.7	97.8	98.0	98.7	98.7	98.5	99.0	98.8	99.2	99.0	98.9	98.9	98.9	99.2	99.2	99.0	99.0		99.6	99.6	99.6	99.6	99.6	97.5	96.6	97.4	97.1	96.0
32	G/4415/2022 Belgium_2022	97.1	97.2	96.8	97.1	97.0	98.9	97.1	96.8	96.8	96.5	96.8	96.9	96.6	95.7	97.7	98.0	98.6	98.5	98.4	99.1	98.9	99.2	99.1	98.9	98.9	99.0	99.1	99.1	99.1	99.5	99.1		100	100	100	100	97.7	96.7	97.4	97.3	96.1
33	G/4416/2022 Belgium_2022	97.1	97.2	96.8	97.1	97.0	98.9	97.1	96.8	96.8	96.5	96.8	96.9	96.6	95.7	97.7	98.0	98.6	98.5	98.4	99.1	98.9	99.2	99.1	98.9	98.9	99.0	99.1	99.1	99.1	99.5	99.1	100		100	100	100	97.7	96.7	97.4	97.3	96.1
34	G/4418/2022 Belgium_2022	97.1	97.2	96.8	97.1	97.0	98.9	97.1	96.8	96.8	96.5	96.8	96.9	96.6	95.7	97.7	98.0	98.6	98.5	98.4	99.1	98.9	99.2	99.1	98.9	98.9	99.0	99.1	99.1	99.1	99.5	99.1	100	100		100	100	97.7	96.7	97.4	97.3	96.1
35	G/4419/2022 Belgium_2022	97.1	97.2	96.8	97.1	97.0	98.9	97.1	96.8	96.8	96.5	96.8	96.9	96.6	95.7	97.7	98.0	98.6	98.5	98.4	99.1	98.9	99.2	99.1	98.9	98.9	99.0	99.1	99.1	99.1	99.5	99.1	100	100	100		100	97.7	96.7	97.4	97.3	96.1
36	G/4420/2022 Belgium_2022	97.1	97.2	96.8	97.1	97.0	98.9	97.1	96.8	96.8	96.5	96.8	96.9	96.6	95.7	97.7	98.0	98.6	98.5	98.4	99.1	98.9	99.2	99.1	98.9	98.9	99.0	99.1	99.1	99.1	99.5	99.1	100	100	100	100		97.7	96.7	97.4	97.3	96.1
37	DQ811784.2 USA_1983	98.6	98.8	98.1	98.7	98.6	97.4	98.8	98.2	98.3	98.1	98.2	98.5	97.9	96.7	98.5	98.2	98.2	98.1	97.9	97.8	97.7	97.5	97.5	97.5	97.4	97.3	97.7	97.7	97.5	97.5	97.6	97.6	97.6	97.6	97.6	97.6		97.1	98.2	98.2	97.7
38	OP037385.1 USA_2017	97.3	97.5	97.0	97.4	97.3	96.7	98.0	98.0	98.2	96.8	98.1	97.2	98.2	96.6	97.5	97.5	97.5	97.3	97.3	97.0	96.9	96.7	96.8	96.7	96.6	96.7	96.9	96.9	96.8	96.8	96.8	96.9	96.9	96.9	96.9	96.9	98.0		98.2	98.2	96.0
39	OP037412.1 USA_2022	97.4	97.5	97.0	97.5	97.3	96.4	98.4	97.9	98.6	96.8	98.8	97.2	97.8	96.9	97.4	97.2	97.2	97.1	97.0	96.8	96.7	96.5	96.5	96.6	96.5	96.3	96.7	96.7	96.5	96.5	96.6	96.6	96.6	96.6	96.6	96.6	98.0	98.2		98.5	96.8
40	HE616739.1 Cuba_2010	97.6	97.8	97.2	97.7	97.6	96.7	98.6	98.4	99.0	97.1	99.2	97.5	98.2	97.4	97.7	97.6	97.5	97.4	97.3	97.1	97.0	96.8	96.8	96.9	96.8	96.6	96.9	96.9	96.9	96.8	96.9	96.9	96.9	96.9	96.9	96.9	98.4	98.4	98.9		96.6
41	**BCoV strain CA25**	99.4	99.6	98.2	99.6	99.5	96.9	98.2	97.3	97.4	98.8	97.4	99.4	97.2	96.0	98.1	97.7	97.8	97.6	97.3	97.2	97.1	96.9	97.0	96.9	96.9	96.8	97.1	97.1	97.0	97.0	97.1	97.0	97.0	97.0	97.0	97.0	98.6	97.3	97.3	97.6	

Figure S1A: A heatmap of nucleotide (lower left segment) and amino acid (upper right segment) sequence identities of BCoV S protein of isolates circulating in Belgium and The Netherlands from 2020 to 2022 compared to other sequences retrieved from GenBank. The colour scale indicates the percent nucleotide (lower left) and aa (upper right) identities. Vaccine strains are in bold, classical strains are shaded in orange, African strain in grey, Asian strains in turquoise, European strains in blue and strains from the Americas in pink. The strains obtained in this study are highlighted in red text.

#	BCoV strains/isolates	1	2	3	4	5	6	7	8	9	10	11	12	13	14	15	16	17	18	19	20	21	22	23	24	25	26	27	28	29	30	31	32	33	34	35	36	37	38	39	40	41
1	**U00735.2 USA_1972**		99.3	99.8	97.0	97.2	97.9	97.4	99.5	98.1	97.2	97.4	97.9	98.8	97.7	97.9	97.9	98.1	98.8	97.2	97.9	98.1	97.9	97.0	97.9	96.7	96.7	97.7	97.9	97.2	97.9	97.7	97.9	97.9	97.9	99.3	98.4	98.4	98.4	98.1	98.4	99.8
2	AB354579.1 Japan_1976	99.8		99.1	96.3	96.5	97.2	96.7	98.8	97.4	96.5	96.7	97.2	98.1	97.0	97.7	97.2	97.4	98.1	96.5	97.2	97.4	97.2	96.7	97.2	96.0	96.0	97.0	97.2	96.5	97.2	97.0	97.2	97.2	97.2	98.6	97.7	97.7	97.7	97.4	97.7	99.1
3	AF220295.1 Canada_1999	99.9	99.7		96.7	97.0	97.7	97.2	99.3	97.9	97.0	97.2	97.7	98.6	97.4	97.7	97.7	97.9	98.6	97.0	97.7	97.9	97.7	96.7	97.7	96.5	96.5	97.4	97.7	97.0	97.7	97.4	97.7	97.7	97.7	99.1	98.1	98.1	98.1	97.9	98.1	99.5
4	PV061390.1 Ethiopia_2023	96.8	96.6	96.7		96.3	96.7	97.2	96.5	96.7	96.5	97.2	97.0	97.7	97.0	96.7	97.0	97.2	97.7	97.9	98.4	98.4	98.6	98.4	98.6	98.8	98.8	98.4	98.6	98.8	98.6	98.4	98.6	98.6	98.6	97.7	97.2	97.2	97.9	97.0	97.0	97.0
5	LC494173.1 Japan_2008	97.4	97.1	97.3	96.7		97.9	97.9	96.7	97.0	96.3	96.5	97.0	98.4	97.7	97.4	97.4	98.1	97.9	96.3	96.7	97.2	97.0	96.0	97.0	96.0	96.0	97.0	97.0	96.3	97.0	96.7	97.0	97.0	97.0	97.9	97.9	97.9	98.4	97.7	97.7	97.2
6	LC494140.1 Japan_2015	97.4	97.2	97.4	96.8	98.8		97.7	97.4	97.7	97.0	97.2	97.7	99.1	97.9	98.1	98.1	98.4	98.6	97.0	97.4	97.9	97.7	96.7	97.7	96.5	96.5	97.7	97.7	97.0	97.7	97.4	97.7	97.7	97.7	98.6	98.6	98.6	98.6	98.4	98.4	97.7
7	LC830477.1 Japan_2022	97.1	96.9	97.0	96.6	98.8	98.4		97.4	97.4	96.7	97.0	97.4	98.6	97.7	97.7	97.9	97.9	98.1	97.2	97.2	97.7	97.4	97.0	97.4	97.0	97.0	97.4	97.4	97.2	97.4	97.2	97.4	97.4	97.4	98.1	98.1	98.6	98.6	98.4	98.4	97.4
8	OP296992.1 Taiwan_2017	99.8	99.6	99.8	96.7	97.2	97.3	97.1		97.7	96.7	97.0	97.7	98.4	97.2	97.4	97.4	97.7	98.4	96.7	97.4	97.7	97.4	96.5	97.4	96.3	96.3	97.2	97.4	96.7	97.4	97.2	97.4	97.4	97.4	98.8	97.9	97.9	97.9	97.7	97.9	99.3
9	KU886219.1 China_2015	97.5	97.3	97.4	96.9	97.8	97.7	97.7	97.4		97.9	97.9	97.4	98.6	98.4	97.7	98.6	97.9	98.4	97.0	97.2	97.7	97.4	96.7	97.4	96.5	96.5	97.4	97.4	97.0	97.4	97.2	97.4	97.4	97.4	98.4	98.4	98.1	98.1	97.9	97.9	97.9
10	MK095137.1 China_2017	96.7	96.4	96.6	96.1	96.7	96.7	96.6	96.5	97.9		98.4	97.4	97.9	98.1	97.0	97.9	97.7	97.9	96.7	97.0	97.2	97.2	96.5	97.2	96.3	96.3	97.0	97.2	96.7	97.2	97.0	97.2	97.2	97.2	97.9	98.1	97.4	97.4	97.2	97.2	97.0
11	MN982193.1 China_2018	96.7	96.5	96.7	96.2	96.7	96.7	96.5	96.6	97.7	99.0		97.7	98.1	98.4	97.2	98.1	97.9	98.1	97.0	97.2	97.4	97.4	96.7	97.4	96.5	96.5	97.2	97.4	97.0	97.4	97.2	97.4	97.4	97.4	98.1	97.9	97.7	97.7	97.4	97.4	97.2
12	PP273504.1 China_2019	97.6	97.4	97.5	96.8	97.8	97.7	97.6	97.4	97.7	97.4	97.3		98.6	97.9	97.7	97.7	98.4	98.6	97.2	97.7	97.9	97.9	97.0	97.9	96.7	96.7	97.7	97.9	97.2	97.9	97.7	97.9	97.9	97.9	98.6	98.1	98.1	98.1	97.9	97.9	97.7
13	OP762501.1 China_2021	97.4	97.2	97.4	97.0	98.1	98.4	97.8	97.3	97.9	96.7	96.7	97.6		98.8	99.1	99.1	99.3	99.5	97.9	98.4	98.8	98.6	97.7	98.6	97.4	97.4	98.6	98.6	97.9	98.6	98.4	98.6	98.6	98.6	99.5	99.5	99.5	99.5	99.3	99.3	98.6
14	OR621174.1 China_2022	97.1	96.9	97.0	96.6	97.6	97.5	97.3	97.0	98.7	98.5	98.6	97.6	97.7		97.9	98.8	99.1	98.4	97.7	97.4	97.7	97.7	97.0	97.7	96.7	96.7	97.7	97.7	97.2	97.7	97.4	97.7	97.7	97.7	98.4	98.4	98.4	98.4	98.1	98.1	97.4
15	OR753439.1 China_2023	97.7	97.7	97.7	97.1	98.5	98.4	98.3	97.6	98.2	97.0	96.9	98.1	98.4	97.8		98.1	98.4	98.6	97.4	97.4	97.9	97.7	97.2	97.7	96.5	96.5	97.7	97.7	97.0	97.7	97.4	97.7	97.7	97.7	98.6	98.6	98.6	98.6	98.4	98.4	97.7
16	PP101630.1 China_2023	97.3	97.0	97.2	96.8	97.6	97.7	97.4	97.1	98.5	97.9	98.0	97.1	98.0	98.8	98.0		98.4	98.6	97.2	97.4	97.9	97.7	97.0	97.7	96.7	96.7	97.7	97.7	97.2	97.7	97.4	97.7	97.7	97.7	98.6	98.6	98.6	98.6	98.4	98.4	97.7
17	PQ588962.1 China_2024	97.9	97.7	97.8	97.2	98.7	98.4	98.3	97.7	98.5	97.6	97.5	98.7	98.4	98.6	98.9	98.0		98.8	97.4	97.9	98.1	98.1	97.2	98.1	97.0	97.0	98.1	98.1	97.4	98.1	97.9	98.1	98.1	98.1	98.8	98.8	98.8	98.8	98.6	98.6	97.9
18	MG757141.1 France_2014	97.9	97.7	97.8	98.1	97.8	97.9	97.7	97.7	97.7	97.0	97.0	97.9	98.0	97.4	98.2	97.7	98.2		98.4	98.4	99.3	98.6	98.1	98.6	97.9	97.9	98.8	98.6	97.9	99.1	98.8	99.1	99.1	99.1	99.5	99.1	99.1	99.1	98.8	98.8	98.6
19	ON792959.1 Ireland_2019	97.4	97.2	97.4	97.6	97.4	97.4	97.1	97.3	97.3	96.7	96.7	97.5	97.6	97.4	97.9	97.4	97.8	98.8		98.1	98.6	98.4	98.1	98.4	98.1	98.1	98.6	98.4	98.1	98.8	98.6	98.8	98.8	98.8	97.9	97.4	97.4	97.4	97.2	97.2	97.0
20	G/639/2020 Belgium_2020	97.3	97.0	97.2	97.8	97.0	97.0	96.8	97.1	96.9	96.3	96.2	97.1	97.1	96.7	97.4	97.0	97.5	98.2	97.9		99.1	99.8	98.1	99.3	98.1	98.1	99.1	99.8	99.1	99.3	99.1	99.3	99.3	99.3	98.4	97.9	97.9	98.4	97.7	98.1	97.7
21	G/640/2020 Belgium_2020	97.6	97.4	97.5	98.1	97.5	97.4	97.3	97.4	97.4	96.7	96.6	97.5	97.6	97.1	97.9	97.4	97.9	98.8	98.4	99.2		99.3	98.6	99.3	98.6	98.6	99.5	99.3	98.6	99.8	99.5	99.8	99.8	99.8	98.8	98.4	98.4	98.8	98.1	98.1	97.9
22	G/666/2020 Belgium_2020	97.6	97.4	97.5	98.3	97.7	97.6	97.4	97.4	97.4	96.7	96.7	97.7	97.6	97.2	98.1	97.4	98.1	98.5	98.2	99.2	99.2		98.4	99.5	98.4	98.4	99.3	100	99.3	99.5	99.3	99.5	99.5	99.5	98.6	98.1	98.1	98.6	97.9	97.9	97.7
23	G/3199/2021 Belgium_2021	97.0	96.9	96.9	98.7	96.9	97.0	96.7	96.8	97.0	96.5	96.4	97.0	97.0	96.9	97.4	97.0	97.4	98.6	98.1	97.9	98.4	98.2		98.4	98.6	98.6	98.6	98.4	98.6	98.8	98.6	98.8	98.8	98.8	97.7	97.2	97.2	97.7	97.0	97.0	96.7
24	G/3202/2022 The Netherlands_2022	97.5	97.3	97.4	98.4	97.4	97.4	97.2	97.4	97.3	96.7	96.6	97.5	97.5	97.1	97.8	97.4	97.9	98.6	98.3	99.3	99.6	99.5	98.3		98.4	98.4	99.3	99.5	98.8	99.5	99.3	99.5	99.5	99.5	98.6	98.1	98.1	98.6	97.9	97.9	97.7
25	G/3662/2022 Belgium_2022	96.7	96.4	96.6	98.5	96.6	96.7	96.4	96.5	96.7	96.0	96.0	96.7	96.7	96.4	97.0	96.7	97.0	98.4	98.0	97.9	98.1	98.1	98.9	98.0		100	98.6	98.4	98.6	98.8	98.6	98.8	98.8	98.8	97.4	97.0	97.0	97.7	96.7	96.7	96.7
26	G/3667/2022 Belgium_2022	96.7	96.4	96.6	98.5	96.6	96.7	96.4	96.5	96.7	96.0	96.0	96.7	96.7	96.4	97.0	96.7	97.0	98.4	98.0	97.9	98.1	98.1	98.9	98.0	100		98.6	98.4	98.6	98.8	98.6	98.8	98.8	98.8	97.4	97.0	97.0	97.7	96.7	96.7	96.7
27	G/4367/2022 Belgium_2022	97.4	97.1	97.3	98.1	97.4	97.3	97.1	97.2	97.2	96.5	96.4	97.4	97.4	97.0	97.7	97.3	97.8	98.6	98.3	99.1	99.8	99.1	98.3	99.5	98.0	98.0		99.3	98.6	99.8	99.5	99.8	99.8	99.8	98.4	98.1	98.1	98.6	97.9	97.9	97.4
28	G/4381/2022 Belgium_2022	97.2	97.0	97.1	97.9	97.1	97.0	96.9	97.0	97.1	96.5	96.4	97.4	97.2	97.0	97.5	97.0	97.9	98.3	98.0	99.5	99.3	99.3	98.0	99.4	97.8	97.8	99.2		99.3	99.5	99.3	99.5	99.5	99.5	98.6	98.1	98.1	98.6	97.9	97.9	97.7
29	G/4382/2022 Belgium_2022	97.3	97.0	97.2	98.8	97.2	97.1	97.0	97.1	97.2	96.6	96.5	97.3	97.3	97.0	97.6	97.3	97.7	98.4	98.1	98.6	98.6	98.9	98.8	98.7	98.7	98.7	98.5	98.7		98.8	98.6	98.8	98.8	98.8	97.9	97.4	97.4	97.9	97.2	97.2	97.0
30	G/4415/2022 Belgium_2022	97.6	97.4	97.5	98.3	97.5	97.4	97.3	97.4	97.4	96.7	96.7	97.6	97.6	97.2	97.9	97.4	98.0	98.8	98.5	99.2	99.7	99.4	98.5	99.6	98.2	98.2	99.6	99.3	98.9		99.8	100	100	100	98.6	98.1	98.1	98.6	97.9	97.9	97.7
31	G/4416/2022 Belgium_2022	97.5	97.3	97.4	98.2	97.4	97.4	97.2	97.4	97.3	96.7	96.6	97.5	97.5	97.1	97.8	97.4	97.9	98.8	98.4	99.1	99.6	99.3	98.4	99.5	98.1	98.1	99.5	99.2	98.8	99.9		99.8	99.8	99.8	98.4	97.9	97.9	98.4	97.7	97.7	97.4
32	G/4418/2022 Belgium_2022	97.6	97.4	97.5	98.3	97.5	97.4	97.3	97.4	97.4	96.7	96.7	97.6	97.6	97.2	97.9	97.4	98.0	98.8	98.5	99.2	99.7	99.4	98.5	99.6	98.2	98.2	99.6	99.3	98.9	100	99.9		100	100	98.6	98.1	98.1	98.6	97.9	97.9	97.7
33	G/4419/2022 Belgium_2022	97.6	97.4	97.5	98.3	97.5	97.4	97.3	97.4	97.4	96.7	96.7	97.6	97.6	97.2	97.9	97.4	98.0	98.8	98.5	99.2	99.7	99.4	98.5	99.6	98.2	98.2	99.6	99.3	98.9	100	99.9	100		100	98.6	98.1	98.1	98.6	97.9	97.9	97.7
34	G/4420/2022 Belgium_2022	97.6	97.4	97.5	98.3	97.5	97.4	97.3	97.4	97.4	96.7	96.7	97.6	97.6	97.2	97.9	97.4	98.0	98.8	98.5	99.2	99.7	99.4	98.5	99.6	98.2	98.2	99.6	99.3	98.9	100	99.9	100	100		98.6	98.1	98.1	98.6	97.9	97.9	97.7
35	DQ811784.2 USA_1983	98.7	98.4	98.6	97.1	98.2	98.3	98.0	98.5	98.1	97.1	97.2	98.2	98.1	97.7	98.4	97.7	98.6	98.2	97.7	97.4	97.9	97.9	97.3	97.8	97.0	97.0	97.7	97.5	97.6	97.9	97.8	97.9	97.9	97.9		99.1	99.1	99.1	98.8	98.8	99.1
36	MH043952.1 USA_2017	97.7	97.5	97.7	97.4	98.3	98.4	98.2	97.6	98.3	97.1	96.9	97.9	98.4	97.7	98.6	98.0	98.6	98.3	97.7	97.4	97.9	97.9	97.5	97.8	97.2	97.2	97.7	97.5	97.6	97.9	97.8	97.9	97.9	97.9	98.4		99.1	99.1	98.8	98.8	98.1
37	OP037418.1 USA_2018	97.3	97.0	97.2	96.9	97.7	97.7	97.8	97.1	97.7	96.4	96.3	97.4	98.3	97.2	98.1	97.4	98.1	97.7	97.2	97.1	97.4	97.4	97.0	97.4	96.8	96.8	97.3	97.0	97.1	97.4	97.4	97.4	97.4	97.4	98.0	98.7		99.1	99.3	99.3	98.1
38	OP037400.1 USA_2020	97.6	97.4	97.5	97.2	98.4	98.2	98.3	97.4	98.1	96.8	96.7	97.7	98.6	97.7	98.6	97.8	98.6	98.1	97.6	97.4	97.9	97.9	97.4	97.8	97.2	97.2	97.7	97.5	97.6	97.9	97.8	97.9	97.9	97.9	98.3	98.7	98.6		98.8	98.8	98.4
39	OR502441.1 USA_2021	97.9	97.7	97.8	97.0	98.4	98.4	98.4	97.7	98.1	97.0	97.0	98.0	98.5	97.9	98.8	98.1	98.8	98.1	97.7	97.5	97.8	98.0	97.2	97.7	96.9	96.9	97.7	97.4	97.5	97.8	97.7	97.8	97.8	97.8	98.4	98.7	98.5	98.5		99.1	97.9
40	OP037436.1 USA_2022	97.4	97.2	97.4	96.7	98.1	98.1	98.0	97.3	97.7	96.7	96.6	97.6	98.3	97.4	98.4	97.5	98.3	97.7	97.4	97.1	97.4	97.4	96.8	97.4	96.6	96.6	97.3	97.0	97.1	97.4	97.4	97.4	97.4	97.4	98.1	98.2	98.0	98.3	98.4		98.1
41	**BCoV strain CA25**	99.9	99.7	99.8	96.8	97.3	97.4	97.0	99.8	97.4	96.6	96.7	97.5	97.4	97.0	97.7	97.2	97.8	97.8	97.4	97.2	97.5	97.5	96.9	97.4	96.7	96.7	97.3	97.1	97.2	97.5	97.4	97.5	97.5	97.5	98.6	97.7	97.2	97.5	97.8	97.4	

Figure S1B: A heatmap of nucleotide (lower left segment) and amino acid (upper right segment) sequence identities of BCoV HE protein of isolates circulating in Belgium and The Netherlands from 2020 to 2022 compared to other sequences retrieved from GenBank. The colour scale indicates the percent nucleotide (lower left) and aa (upper right) identities. Vaccine strains are shown in bold, classical strains are shaded in orange, African strain in grey, Asian strains in turquoise, European strains in blue and strains from the Americas in pink. The strains obtained in this study are highlighted in red text.

#	PHEV strains/isolates	1	2	3	4	5	6	7	8	9	10	11	12	13	14	15	16	17	18	19	20	21	22	23	24	25	26	27	28	29	30	31	32	33	34	35	36	37	38	39	40
1	MW165134.1 USA_1970		98.3	97.6	98.4	97.3	97.1	96.6	97.2	96.4	96.4	97.3	97.3	96.9	96.4	96.8	96.8	97.2	96.1	96.4	97.0	96.5	96.6	96.1	96.4	96.5	96.4	96.6	96.7	96.9	96.4	96.4	96.7	96.4	98.3	96.6	96.2	97.0	97.1	97.0	97.1
2	DQ011855.1 Belgium_1972	97.9		97.1	98.1	96.9	97.0	96.2	97.0	96.0	96.0	97.1	97.1	96.8	96.4	96.7	96.2	96.7	95.9	96.0	96.8	96.1	96.2	95.8	96.0	96.3	96.3	96.2	96.4	96.6	96.5	96.4	96.4	97.1	99.9	97.0	96.6	96.7	96.7	96.7	96.8
3	OL542832.1 South_Korea_2021	97.5	96.7		97.0	96.1	96.2	95.5	96.3	95.1	95.0	96.4	96.4	96.0	95.8	96.0	95.9	96.1	95.1	95.1	96.1	95.6	95.7	95.3	95.4	95.6	95.4	95.9	95.8	95.7	95.6	95.4	95.6	95.3	97.0	95.3	95.0	96.2	95.9	95.9	95.9
4	KY994645.1 China_2008	98.6	98.0	97.4		96.8	97.0	96.1	97.0	95.8	95.8	97.2	97.2	96.7	96.3	96.7	96.4	96.8	95.9	95.8	96.8	96.0	96.1	95.8	95.9	96.0	96.0	96.1	96.5	96.3	96.4	96.3	96.4	96.1	98.1	96.4	95.9	96.7	96.6	96.7	96.6
5	OQ798825.1 China_2016	97.0	96.4	95.9	97.1		98.2	95.7	98.2	95.5	95.9	98.6	98.4	98.1	97.4	97.8	97.2	98.6	96.7	95.5	98.0	97.5	97.2	96.8	97.0	97.1	96.7	97.2	97.8	96.1	97.1	97.4	97.5	95.4	96.7	95.3	95.0	96.4	95.9	96.2	96.1
6	OQ305205.1 China_2017	97.1	96.6	95.9	97.3	98.3		95.3	99.0	95.1	95.4	99.1	99.0	98.7	98.1	98.5	96.8	98.4	97.6	95.1	98.9	97.7	97.3	97.6	97.1	97.3	96.5	97.3	98.5	95.8	97.7	98.0	98.1	95.1	96.8	95.3	94.9	96.3	95.6	95.9	95.8
7	OQ305206.1 China_2017	97.1	96.5	95.8	96.9	96.2	96.1		95.4	99.3	97.5	95.6	95.7	95.2	95.0	95.3	95.2	95.6	94.4	99.3	95.2	94.9	95.0	94.3	94.8	95.0	96.5	95.0	95.2	96.7	96.3	95.7	95.0	94.6	96.1	95.3	94.5	95.8	95.7	96.0	96.1
8	OQ305207.1 China_2017	97.1	96.4	96.0	97.3	98.4	98.8	96.1		95.2	95.3	99.1	99.0	98.7	98.3	98.5	96.7	98.3	97.6	95.2	98.7	97.7	97.5	97.5	97.3	97.4	96.4	97.5	98.5	95.9	97.8	97.9	98.1	95.0	96.8	95.2	95.0	96.3	95.6	95.9	95.9
9	OQ798806.1 China_2018	96.8	96.2	95.5	96.6	96.0	95.8	99.5	95.9		97.2	95.3	95.5	95.0	94.7	95.0	95.0	95.3	94.2	99.3	95.0	94.7	94.7	94.2	94.6	94.7	96.2	94.7	95.0	96.4	96.0	95.4	94.7	94.4	95.9	95.0	94.3	95.6	95.7	95.8	95.9
10	OQ798810.1 China_2019	97.1	96.4	96.0	96.9	96.2	96.1	97.6	96.1	97.3		95.6	95.6	95.1	94.7	95.0	95.3	95.5	94.5	97.2	95.2	95.0	94.9	94.2	94.7	94.8	96.7	94.9	95.1	97.3	95.9	95.6	94.7	94.6	95.9	95.2	94.7	96.0	95.7	96.1	96.1
11	OQ798832.1 China_2020	97.0	96.5	95.9	97.2	98.7	98.9	96.2	98.8	95.9	96.0		99.6	99.3	98.3	98.7	96.8	98.5	97.6	95.3	98.8	97.6	97.4	97.6	97.2	97.3	96.7	97.4	98.7	96.1	97.9	98.1	98.2	95.2	97.0	95.3	95.1	96.4	95.8	96.1	95.9
12	OQ798833.1 China_2020	97.0	96.4	95.9	97.2	98.4	98.8	96.1	98.7	95.9	96.0	99.4		99.4	98.4	98.7	96.7	98.4	97.6	95.5	98.9	97.5	97.3	97.7	97.0	97.2	96.7	97.3	98.6	96.1	97.9	98.1	98.3	95.3	97.0	95.4	95.2	96.4	95.8	96.1	96.1
13	OQ798826.1 China_2020	96.8	96.2	95.6	96.9	98.0	98.6	95.8	98.5	95.5	95.7	99.3	99.3		97.9	98.3	96.4	98.1	97.3	95.0	98.5	97.2	97.0	97.3	96.7	96.9	96.2	97.0	98.3	95.6	97.6	97.7	97.9	94.9	96.7	95.0	94.8	95.9	95.3	95.6	95.6
14	PP646298.1 China_2021	96.8	96.2	95.9	97.0	98.0	98.5	95.9	98.5	95.6	95.7	98.4	98.3	98.1		99.0	95.8	97.5	96.7	94.7	98.0	96.6	96.4	96.9	96.1	96.4	95.7	96.4	97.7	95.2	97.5	97.0	97.4	94.3	96.2	94.5	94.2	95.6	94.9	95.2	95.1
15	PP646299.1 China_2021	96.9	96.3	96.0	97.1	98.0	98.6	95.9	98.5	95.7	95.8	98.5	98.4	98.1	99.6		96.1	97.9	97.0	95.0	98.4	97.0	96.7	97.2	96.5	96.8	96.1	96.7	98.1	95.5	97.9	97.4	97.8	94.7	96.6	94.9	94.6	95.9	95.3	95.6	95.5
16	OQ798807.1 China_2021	96.9	96.3	95.8	97.1	96.8	96.5	95.8	96.4	95.5	95.9	96.6	96.4	96.1	96.2	96.3		97.0	95.8	95.0	96.4	96.2	96.1	95.2	95.9	96.1	95.9	96.1	96.2	95.6	95.8	96.1	95.7	94.9	96.1	95.0	94.7	96.2	95.7	95.9	95.6
17	OQ798809.1 China_2021	97.1	96.5	95.8	97.2	98.6	98.7	96.2	98.3	96.0	96.1	98.5	98.4	98.2	98.0	98.1	96.8		97.0	95.3	98.1	97.6	97.4	96.9	97.3	97.3	96.6	97.4	98.0	96.1	97.0	97.5	97.6	95.0	96.6	95.1	94.8	96.4	95.9	96.1	95.9
18	OQ798818.1 China_2021	96.7	96.1	95.6	96.8	97.4	97.8	95.7	97.9	95.5	95.6	97.7	97.7	97.5	97.5	97.5	96.1	97.6		94.3	97.3	96.2	96.0	96.6	95.8	95.9	95.6	96.0	97.2	94.8	96.7	96.5	96.9	94.2	95.7	94.4	94.3	95.3	94.7	95.1	94.7
19	PP646303.1 China_2022	96.7	96.2	95.4	96.5	95.8	95.7	99.3	95.7	99.3	97.2	95.8	95.8	95.4	95.4	95.5	95.4	95.8	95.4		95.0	94.7	94.7	94.1	94.6	94.7	96.2	94.7	95.0	96.4	96.1	95.4	94.7	94.4	95.9	95.2	94.3	95.6	95.5	95.9	95.9
20	PP646304.1 China_2022	96.9	96.3	95.8	97.0	98.1	98.8	95.9	98.5	95.7	95.8	98.6	98.5	98.3	98.3	98.3	96.2	98.3	97.6	95.5		97.3	97.0	97.5	96.8	97.0	96.2	97.0	98.2	95.6	97.4	97.6	98.1	95.0	96.7	95.0	94.7	96.1	95.4	95.7	95.6
21	PP646305.1 China_2022	96.4	95.8	95.3	96.4	97.4	97.3	95.1	97.4	94.8	95.3	97.2	97.1	96.9	96.8	96.9	95.9	97.4	96.3	94.7	97.2		98.7	96.1	98.5	98.7	96.0	98.7	97.3	95.6	96.5	96.8	96.7	94.8	96.0	94.5	94.3	95.8	95.3	95.4	95.3
22	PP646307.1 China_2022	96.4	95.6	95.3	96.2	97.0	96.9	95.2	97.1	94.9	95.2	96.8	96.7	96.5	96.5	96.6	95.8	97.0	96.0	94.8	96.7	98.9		95.9	99.3	99.5	95.9	99.6	96.9	95.4	96.6	96.6	96.5	94.9	96.1	94.6	94.4	95.8	95.4	95.4	95.6
23	PP646311.1 China_2022	96.8	96.1	95.7	96.9	97.7	98.4	95.6	98.1	95.3	95.6	98.2	98.2	98.0	97.8	97.9	96.0	97.8	97.4	95.2	98.1	96.8	96.4		95.8	95.9	95.3	95.9	97.3	94.7	96.4	96.4	97.3	94.3	95.6	94.1	94.1	95.3	94.3	94.7	94.5
24	PP646317.1 China_2023	96.2	95.5	95.1	96.1	96.9	96.9	95.0	97.0	94.7	95.1	96.8	96.6	96.4	96.5	96.6	95.7	97.0	95.9	94.6	96.7	98.8	99.5	96.3		99.3	95.6	99.3	96.7	95.3	96.4	96.4	96.4	94.7	95.9	94.4	94.1	95.6	95.3	95.2	95.4
25	PP646318.1 China_2023	96.3	95.6	95.2	96.2	97.0	96.8	95.1	97.0	94.8	95.1	96.8	96.6	96.5	96.5	96.6	95.7	97.0	96.0	94.7	96.7	98.9	99.6	96.4	99.5		95.8	99.5	96.8	95.3	96.7	96.6	96.4	94.8	96.1	94.6	94.3	95.7	95.3	95.3	95.6
26	PP646320.1 China_2023	96.8	96.3	95.7	96.9	97.3	97.4	96.6	97.2	96.3	97.0	97.3	97.1	96.9	96.8	96.9	96.1	97.5	96.5	96.2	97.0	96.3	95.9	96.7	95.8	95.9		95.9	96.1	96.7	96.9	97.0	96.0	94.6	96.1	94.7	94.8	95.9	95.2	96.2	95.9
27	PP646323.1 China_2023	96.4	95.7	95.3	96.3	97.1	96.9	95.1	97.1	94.9	95.2	96.9	96.7	96.6	96.5	96.6	95.8	97.1	96.1	94.8	96.8	98.9	99.7	96.4	99.6	99.7	96.0		96.9	95.4	96.6	96.6	96.5	94.9	96.1	94.6	94.4	95.8	95.4	95.4	95.6
28	PP646326.1 China_2023	96.8	96.2	95.6	96.9	98.0	98.6	95.7	98.4	95.5	95.7	98.4	98.3	98.2	98.0	98.1	96.0	98.2	97.5	95.3	98.4	97.4	96.7	98.0	96.7	96.7	96.9	96.8		95.5	97.4	97.7	97.8	94.6	96.3	95.0	94.6	95.7	95.2	95.5	95.6
29	PP646329.1 China_2023	97.1	96.6	96.2	97.1	96.3	96.3	96.8	96.2	96.6	97.5	96.3	96.1	96.0	96.0	96.1	95.9	96.4	95.8	96.5	96.1	95.7	95.3	95.9	95.2	95.3	97.0	95.4	96.0		96.1	96.1	95.2	95.1	96.4	95.2	94.9	96.7	95.9	96.0	96.5
30	PP646334.1 China_2023	96.9	96.3	96.0	97.0	97.4	97.9	96.6	97.8	96.3	96.5	97.9	97.8	97.6	98.4	98.5	96.2	97.5	97.1	96.3	97.6	96.4	96.5	97.4	96.5	96.5	97.3	96.5	97.6	96.4		97.6	97.0	94.7	96.4	94.9	94.9	95.6	94.9	95.6	95.8
31	PP646347.1 China_2024	96.8	96.1	95.6	96.9	97.6	98.2	96.1	97.9	95.9	96.1	98.0	98.0	97.8	97.7	97.8	96.1	97.8	97.2	95.8	97.9	96.9	96.3	97.4	96.3	96.3	97.3	96.4	98.5	96.4	97.7		97.2	94.7	96.2	95.0	94.9	95.7	95.2	95.8	96.0
32	PV730382.1 China_2025	96.7	96.1	95.6	96.8	97.8	98.4	95.7	98.1	95.4	95.6	98.2	98.1	98.0	97.9	98.0	95.9	98.0	97.3	95.3	98.3	96.9	96.3	98.0	96.3	96.2	96.9	96.3	98.5	96.0	97.4	98.0		94.7	96.3	94.7	94.7	95.5	95.1	95.3	95.3
33	KY492680.1 The_Netherlands_2015	96.5	97.5	95.5	96.5	95.4	95.4	95.2	95.3	95.0	95.0	95.3	95.2	95.0	94.9	95.0	95.3	95.5	94.9	94.9	95.0	94.7	94.7	95.0	94.6	94.7	95.1	94.7	94.8	95.4	95.2	95.0	94.9		97.0	96.4	97.4	95.2	95.0	95.0	95.2
34	G/412/2020 The Netherlands_2020	97.9	100	96.6	98.0	96.4	96.5	96.4	96.4	96.2	96.3	96.4	96.4	96.1	96.2	96.3	96.3	96.4	96.0	96.2	96.2	95.7	95.6	96.1	95.5	95.6	96.2	95.6	96.1	96.6	96.2	96.1	96.0	97.4		96.8	96.4	96.6	96.5	96.6	96.7
35	G/766/2021 The Netherlands_2021	96.2	97.0	95.2	96.4	95.1	95.3	95.2	95.2	95.0	94.9	95.1	95.0	94.9	94.8	94.8	94.9	95.4	94.8	94.9	94.8	94.4	94.3	94.6	94.2	94.3	94.9	94.3	94.8	95.1	94.8	94.9	94.6	97.2	96.9		96.6	95.0	95.5	95.0	95.4
36	G/7664/2023 Austria_2023	96.4	97.2	95.3	96.4	95.3	95.3	95.2	95.3	95.0	95.2	95.2	95.2	95.0	94.9	95.0	95.1	95.4	94.8	95.0	95.0	94.6	94.5	94.9	94.4	94.5	95.1	94.5	94.8	95.2	95.2	95.0	94.9	98.1	97.2	97.1		94.7	94.8	95.0	95.0
37	KY419103.1 USA_2015	97.4	96.7	96.3	97.2	96.4	96.2	96.5	96.3	96.3	96.9	96.2	96.1	95.9	96.0	96.1	96.2	96.4	96.0	96.2	96.1	95.7	95.6	96.0	95.5	95.6	96.2	95.7	95.9	96.9	96.1	96.0	95.8	95.5	96.6	95.1	95.4		96.0	96.4	96.4
38	KY419105.1 USA_2015	97.5	96.8	96.5	97.4	96.5	96.2	96.6	96.4	96.4	96.9	96.2	96.2	95.9	95.9	96.0	96.3	96.5	95.8	96.3	96.1	95.8	95.8	95.8	95.7	95.7	96.2	95.8	95.8	96.6	96.0	95.9	95.9	95.6	96.8	95.5	95.4	97.0		96.3	96.7
39	KY419113.1 USA_2015	97.3	96.8	96.5	97.3	96.4	96.2	96.5	96.2	96.2	96.8	96.1	96.1	95.8	95.9	96.0	96.0	96.3	95.8	96.1	96.0	95.7	95.7	95.7	95.5	95.7	96.3	95.7	95.8	96.4	96.0	95.9	95.7	95.4	96.7	95.3	95.4	96.9	97.2		96.4
40	PQ241464.1 USA_2021	97.4	96.8	96.4	97.3	96.4	96.1	96.7	96.2	96.4	97.0	96.1	96.1	95.8	95.9	96.0	95.9	96.1	95.7	96.3	96.0	95.5	95.4	95.7	95.3	95.4	96.4	95.5	96.0	97.0	96.2	96.2	95.9	95.4	96.8	95.2	95.3	97.1	97.4	96.9	

Figure S1C: A heatmap of nucleotide (lower left segment) and amino acid (upper right segment) sequence identities of the PHEV S protein of isolates circulating in Austria and the Netherlands from 2020 to 2023 compared to other sequences retrieved from GenBank. The colour scale indicates the percent nucleotide (lower left) and aa (upper right) identities. Classical strains including VW572 (DQ118855.1, Belgium_1972) are shaded in orange; Asian strains in turquoise; European strains in blue; and strains from North America in pink. The strains obtained in this study are highlighted in red text.

#	PHEV strains/isolates	1	2	3	4	5	6	7	8	9	10	11	12	13	14	15	16	17	18	19	20	21	22	23	24	25	26	27	28	29	30	31	32	33	34	35	36	37	38	39	40
1	MW165134.1 USA_1970		95.3	97.9	97.9	97.6	96.0	96.5	96.5	96.7	97.2	97.2	96.9	97.4	97.4	96.7	96.9	96.7	97.9	96.9	97.2	96.9	97.2	97.2	97.2	96.9	97.4	96.7	96.0	96.2	96.2	96.0	93.4	93.9	97.2	97.4	96.7	97.4	96.7	97.4	96.7
2	DQ011855.1 Belgium_1972	97.0		96.0	96.0	95.1	93.9	93.6	93.4	94.1	94.4	94.8	94.6	94.6	94.6	94.4	94.6	94.1	95.3	94.1	94.4	94.1	94.6	94.8	94.4	94.1	94.6	94.1	99.3	93.6	93.4	99.3	95.8	96.0	94.1	95.1	93.4	95.1	93.4	95.1	93.6
3	KY994645.1 China_2008	99.1	97.5		99.5	97.9	96.2	95.8	95.8	96.5	96.9	97.4	97.2	97.2	97.2	96.9	97.2	97.2	98.1	96.9	96.9	96.7	97.4	97.4	96.9	96.7	97.6	97.2	96.7	96.5	95.8	96.7	94.6	95.1	96.5	97.2	96.0	97.2	96.0	97.2	96.0
4	MF083115.1 China_2014	99.1	97.5	99.8		97.9	96.2	96.2	96.2	96.9	97.4	97.4	97.2	97.6	97.6	96.9	97.2	96.9	98.1	96.9	97.4	97.2	97.4	97.4	97.4	97.2	98.1	96.9	96.7	96.5	96.0	96.7	94.6	95.1	96.9	97.4	96.5	97.4	96.5	97.4	96.5
5	OQ798825.1 China_2016	97.5	95.6	97.8	97.8		96.9	95.5	95.5	96.2	96.7	98.1	97.9	96.9	96.9	97.6	97.9	98.6	99.8	98.6	96.7	96.5	98.1	98.1	96.7	96.5	97.9	98.6	95.5	97.2	95.3	95.5	94.1	94.6	96.7	96.9	96.0	96.9	96.0	96.9	96.0
6	OQ305205.1 China_2017	97.0	95.7	97.3	97.3	97.1		94.6	94.6	94.8	95.3	98.4	98.6	95.5	95.5	98.1	98.6	96.0	97.2	96.0	95.3	95.1	98.4	96.7	95.3	95.1	96.5	96.0	94.4	97.4	93.9	94.4	92.9	93.4	95.3	95.3	94.1	95.3	94.1	95.3	94.4
7	OQ305208.1 China_2017	97.4	95.3	97.1	97.3	95.9	95.5		99.5	98.1	98.6	95.3	95.1	98.4	98.4	94.8	95.1	94.6	95.8	95.3	98.1	98.4	95.3	95.1	98.6	98.4	96.0	94.6	94.4	94.6	95.8	94.4	92.0	92.5	97.2	96.0	96.2	96.0	96.2	96.0	96.0
8	OQ798806.1 China_2018	97.6	95.3	97.3	97.4	96.1	95.7	99.7		98.1	98.6	95.3	95.1	98.4	98.4	94.8	95.1	94.6	95.8	95.3	98.1	98.4	95.3	95.1	98.6	98.4	96.0	94.6	94.1	94.6	95.8	94.1	92.0	92.5	97.2	96.0	96.2	96.0	96.2	96.0	96.0
9	OQ798810.1 China_2019	97.4	95.2	97.3	97.4	96.1	95.5	98.4	98.5		99.5	96.5	96.2	99.3	99.3	96.2	96.2	95.3	96.5	95.3	99.1	99.3	96.0	95.8	99.5	99.3	96.9	95.3	94.6	95.3	95.8	94.6	92.5	92.9	97.6	97.2	96.5	97.2	96.5	97.2	96.2
10	OQ798811.1 China_2019	97.6	95.4	97.4	97.6	96.2	95.7	98.4	98.5	99.7		96.5	96.2	99.8	99.8	96.2	96.2	95.8	96.9	95.8	99.5	99.8	96.5	96.2	100	99.8	97.4	95.8	95.1	95.8	96.0	95.1	92.9	93.4	98.1	97.2	96.9	97.2	96.9	97.2	96.7
11	OQ798827.1 China_2019	97.6	96.0	97.9	97.9	97.6	98.5	95.9	96.1	96.2	96.2		99.8	96.7	96.7	99.3	99.8	97.2	98.4	97.2	96.5	96.2	99.5	97.6	96.5	96.2	97.6	97.2	95.1	98.1	94.8	95.1	93.4	93.9	96.5	96.5	95.3	96.5	95.3	96.5	95.5
12	OQ798829.1 China_2019	97.6	96.0	97.9	97.9	97.3	98.7	95.9	96.1	96.2	96.2	99.5		96.5	96.5	99.5	100	96.9	98.1	96.9	96.2	96.0	99.3	97.4	96.2	96.0	97.4	96.9	94.8	98.4	94.6	94.8	93.2	93.6	96.2	96.2	95.1	96.2	95.1	96.2	95.3
13	OQ798812.1 China_2020	97.6	95.4	97.4	97.6	96.2	95.7	98.4	98.5	99.7	99.7	96.2	96.2		99.5	96.5	96.5	96.0	97.2	96.0	99.3	99.5	96.7	96.5	99.8	99.5	97.6	96.0	95.3	96.0	96.2	95.3	92.7	93.2	98.1	97.4	97.2	97.4	97.2	97.4	96.5
14	OQ798817.1 China_2020	97.6	95.5	97.5	97.6	96.4	95.8	98.4	98.5	99.7	99.8	96.4	96.4	99.7		96.5	96.5	96.0	97.2	96.0	99.3	99.5	96.7	96.5	99.8	99.5	97.6	96.0	95.3	96.0	96.2	95.3	93.2	93.6	98.4	97.4	96.7	97.4	96.7	97.4	96.9
15	OQ798826.1 China_2020	97.4	95.8	97.7	97.7	97.1	98.1	95.8	95.9	96.2	96.3	99.0	99.3	96.2	96.3		99.5	96.7	97.9	96.7	96.2	96.0	98.8	97.2	96.2	96.0	97.4	96.7	94.6	97.9	94.8	94.6	92.9	93.4	96.2	96.0	94.8	96.0	94.8	96.0	95.1
16	OQ798828.1 China_2020	97.5	95.9	97.8	97.8	97.3	98.6	96.0	96.2	96.3	96.3	99.5	99.9	96.3	96.5	99.2		96.9	98.1	96.9	96.2	96.0	99.3	97.4	96.2	96.0	97.4	96.9	94.8	98.4	94.6	94.8	93.2	93.6	96.2	96.2	95.1	96.2	95.1	96.2	95.3
17	OQ798807.1 China_2021	96.6	94.7	96.9	96.9	98.2	96.5	95.1	95.2	95.2	95.4	97.0	96.5	95.4	95.5	96.4	96.6		98.8	97.6	95.8	95.5	97.2	97.2	95.8	95.5	96.9	100	94.6	96.7	94.8	94.6	92.9	93.4	95.8	96.0	94.8	96.0	94.8	96.0	95.1
18	OQ798808.1 China_2021	97.6	95.8	98.0	98.0	99.4	97.1	96.1	96.2	96.2	96.4	97.7	97.4	96.4	96.5	97.3	97.5	98.7		98.8	96.9	96.7	98.4	98.4	96.9	96.7	98.1	98.8	95.8	97.4	95.5	95.8	94.1	94.6	96.9	97.2	96.0	97.2	96.0	97.2	96.2
19	OQ798809.1 China_2021	97.0	95.2	97.3	97.3	98.0	96.5	95.6	95.8	95.5	95.7	96.9	96.7	95.7	95.8	96.7	96.8	97.3	98.0		95.8	95.5	97.2	97.2	95.8	95.5	96.9	97.6	94.6	96.2	94.6	94.6	92.9	93.4	96.0	96.0	95.1	96.0	95.1	96.0	95.3
20	OQ798814.1 China_2021	97.9	95.7	97.7	97.9	96.5	96.0	98.2	98.4	99.1	99.2	96.5	96.5	99.1	99.2	96.5	96.6	95.7	96.7	96.0		99.8	96.5	96.2	99.5	99.8	97.4	95.8	95.1	95.8	96.0	95.1	92.9	93.4	97.6	97.2	96.9	97.2	96.9	97.2	96.7
21	OQ798816.1 China_2021	97.4	95.2	97.3	97.4	96.1	95.5	98.4	98.5	99.2	99.2	96.1	96.1	99.2	99.2	96.0	96.2	95.2	96.2	95.5	99.4		96.2	96.0	99.8	100	97.2	95.5	94.8	95.5	95.8	94.8	92.7	93.2	97.9	96.9	96.7	96.9	96.7	96.9	96.5
22	OQ798818.1 China_2021	97.1	95.8	97.4	97.4	96.9	98.2	95.5	95.6	95.6	95.8	98.6	98.6	95.8	95.9	98.0	98.5	96.4	97.1	96.5	96.1	95.6		97.6	96.5	96.2	97.6	97.2	95.1	98.1	94.8	95.1	93.4	93.9	96.5	96.5	95.3	96.5	95.3	96.5	95.5
23	OQ798819.1 China_2021	96.9	95.4	97.3	97.3	96.9	97.2	95.2	95.4	95.4	95.5	97.3	97.2	95.7	95.7	97.0	97.1	96.2	97.1	96.9	95.8	95.5	97.0		96.2	96.0	98.4	97.2	95.3	96.7	95.1	95.3	93.6	94.1	96.2	96.7	95.3	96.7	95.3	96.7	95.5
24	OQ798820.1 China_2021	97.5	95.3	97.3	97.5	96.2	95.6	98.4	98.6	99.3	99.3	96.2	96.2	99.3	99.3	96.1	96.2	95.3	96.3	95.6	99.3	99.9	95.7	95.6		99.8	97.4	95.8	95.1	95.8	96.0	95.1	92.9	93.4	98.1	97.2	96.9	97.2	96.9	97.2	96.7
25	OQ798821.1 China_2021	97.3	95.1	97.2	97.3	96.0	95.5	98.3	98.4	99.1	99.1	96.0	96.0	99.1	99.1	95.9	96.1	95.1	96.2	95.5	99.3	99.9	95.5	95.5	99.8		97.2	95.5	94.8	95.5	95.8	94.8	92.7	93.2	97.9	96.9	96.7	96.9	96.7	96.9	96.5
26	OQ798822.1 China_2021	97.2	95.5	97.5	97.6	96.9	96.8	95.8	96.0	96.2	96.4	97.2	97.0	96.5	96.5	96.9	96.9	96.2	97.0	96.6	96.9	96.5	96.9	98.7	96.6	96.5		96.9	95.3	96.7	95.5	95.3	93.2	93.6	96.9	96.9	96.0	96.9	96.0	96.9	96.0
27	OQ798823.1 China_2021	96.8	94.9	97.1	97.1	98.0	96.6	95.2	95.4	95.4	95.5	97.0	96.7	95.5	95.7	96.5	96.8	99.8	98.5	97.5	95.8	95.4	96.5	96.4	95.5	95.3	96.3		94.6	96.7	94.8	94.6	92.9	93.4	95.8	96.0	94.8	96.0	94.8	96.0	95.1
28	OP959790.1 China_2021	97.3	99.8	97.7	97.7	95.8	95.9	95.5	95.5	95.5	95.6	96.2	96.2	95.6	95.7	96.1	96.2	95.0	96.0	95.5	95.9	95.5	96.0	95.6	95.5	95.4	95.7	95.1		94.1	94.1	100	96.2	96.5	94.6	95.8	94.1	95.8	94.1	95.8	94.1
29	PV730382.1 China_2025	96.6	95.4	96.9	96.9	96.5	97.7	95.2	95.2	95.4	95.5	97.8	98.1	95.5	95.7	97.6	98.0	95.9	96.6	96.1	95.8	95.4	97.5	96.7	95.5	95.3	96.5	96.1	95.6		94.4	94.1	92.5	92.9	95.8	96.0	94.6	96.0	94.6	96.0	94.6
30	OL542832.1 South_Korea_2021	96.6	94.7	96.3	96.5	94.7	94.7	96.1	96.1	95.9	95.9	95.0	95.0	95.9	96.0	95.0	94.9	94.5	94.9	94.6	96.2	95.8	94.5	94.7	95.8	95.7	95.1	94.7	94.9	94.7		94.1	91.3	91.8	95.5	96.0	95.3	96.0	95.3	96.0	96.0
31	G/412/2020 The Netherlands_2020	97.3	99.8	97.7	97.7	95.8	95.9	95.5	95.5	95.5	95.6	96.2	96.2	95.6	95.7	96.1	96.2	95.0	96.0	95.5	95.9	95.5	96.0	95.6	95.5	95.4	95.7	95.1	100	95.6	94.9		96.2	96.5	94.6	95.8	94.1	95.8	94.1	95.8	94.1
32	G/766/2021 The Netherlands_2021	95.4	96.6	95.9	95.9	94.4	94.4	93.6	93.8	93.7	93.9	94.7	94.7	93.9	94.0	94.5	94.6	93.6	94.4	94.0	94.2	93.7	94.6	94.4	93.8	93.6	94.1	93.7	96.9	94.0	92.8	96.9		99.5	92.7	93.2	92.0	93.2	92.0	93.2	92.2
33	G/7664/2023 Austria_2023	95.5	96.7	96.0	96.0	94.5	94.8	93.6	93.9	93.8	94.0	94.9	95.1	94.0	94.0	94.7	95.0	93.8	94.5	94.1	94.3	93.8	95.0	94.4	93.9	93.7	94.2	94.0	96.9	94.3	93.0	96.9	99.6		93.2	93.6	92.5	93.6	92.5	93.6	92.7
34	KY419103.1 USA_2015	97.8	95.6	97.5	97.6	96.2	95.7	97.0	97.0	96.9	97.1	96.2	96.2	96.9	97.2	96.2	96.2	95.5	96.5	95.8	97.3	96.9	96.1	95.8	97.0	96.9	96.3	95.7	95.8	95.6	96.0	95.8	94.3	94.4		96.5	96.7	96.5	96.7	96.5	96.5
35	KY419107.1 USA_2015	98.0	96.0	97.9	98.0	96.5	96.0	96.9	97.1	97.3	97.3	96.9	96.7	97.4	97.3	96.5	96.8	95.9	96.8	95.9	97.6	97.1	96.1	95.9	97.2	97.0	96.5	96.1	96.2	95.9	96.4	96.2	94.5	94.7	96.9		96.2	100	96.2	100	96.2
36	KY419110.1 USA_2015	97.7	95.4	97.4	97.6	96.0	95.6	96.9	97.0	96.9	97.0	96.0	96.0	97.2	97.0	95.8	95.9	95.1	96.2	95.8	97.3	96.9	95.7	95.7	96.9	96.8	96.1	95.2	95.6	95.2	95.7	95.6	94.2	94.3	96.9	97.2		96.2	100	96.2	95.8
37	KY419111.1 USA_2015	98.0	96.0	97.9	98.0	96.5	96.0	96.9	97.1	97.3	97.3	96.9	96.7	97.4	97.3	96.5	96.8	95.9	96.8	95.9	97.6	97.1	96.1	95.9	97.2	97.0	96.5	96.1	96.2	95.9	96.4	96.2	94.5	94.7	96.9	100	97.2		96.2	100	96.2
38	KY419112.1 USA_2015	97.9	95.5	97.6	97.7	96.2	95.8	97.0	97.2	97.0	97.2	96.2	96.2	97.2	97.2	96.0	96.1	95.3	96.3	95.9	97.5	97.0	95.8	95.7	97.1	96.9	96.1	95.4	95.8	95.4	95.8	95.8	94.2	94.3	97.1	97.2	99.8	97.2		96.2	95.8
39	KY419113.1 USA_2015	98.0	96.0	97.9	98.0	96.5	96.0	96.9	97.1	97.3	97.3	96.9	96.7	97.4	97.3	96.5	96.8	95.9	96.8	95.9	97.6	97.1	96.1	95.9	97.2	97.0	96.5	96.1	96.2	95.9	96.4	96.2	94.5	94.7	96.9	100	97.2	100	97.2		96.2
40	PQ241464.1 USA_2021	97.9	95.5	97.6	97.7	96.5	95.7	97.1	97.3	97.1	97.3	96.2	96.2	97.1	97.4	96.1	96.3	95.6	96.6	95.8	97.4	97.1	95.9	95.8	97.2	97.0	96.3	95.8	95.8	95.3	96.1	95.8	94.0	94.1	97.3	97.3	96.9	97.3	97.1	97.3	

Figure S1D: A heatmap of the aa sequence identity of the PHEV HE protein of isolates circulating in Austria and the Netherlands from 2020 to 2023 compared to other sequences retrieved from GenBank. The colour scale indicates the percent nucleotide (lower left) and aa (upper right) identities. Classical strains including VW572 (DQ118855.1, Belgium_1972) are shaded in orange, Asian strains in turquoise, European strains in blue, and strains from North America in pink. The strains obtained in this study are highlighted in red text.

#	PRCV strains/isolates	**1**	2	3	4	5	6	7	8	9	**10**	**11**	**12**	13	14	15	16	17	18	19	20	21	22	23	24	25	26	27	28	29	30	31	32	33	34	35	36	37	38	39	40
1	**PRCV isolateTLM83_Belgium_1984**		99.4	99.3	97.5	97.5	97.5	97.4	97.1	96.7	99.2	99.2	98.8	99.2	98.6	97.9	96.7	96.8	96.9	96.7	96.7	96.8	96.8	96.7	96.7	96.7	96.7	96.6	96.7	96.7	96.7	96.7	96.7	96.5	97.4	97.3	97.4	96.5	96.3	95.3	95.6
2	OM830318.1 UK_1986	99.6		99.0	97.1	97.1	97.2	97.1	96.8	96.4	99.0	99.0	98.7	99.0	98.4	97.8	96.6	96.7	96.7	96.5	96.6	96.7	96.7	96.5	96.5	96.5	96.5	96.4	96.5	96.6	96.6	96.6	96.5	96.3	97.1	97.0	97.1	96.2	95.9	94.8	95.3
3	M94097.1 The_Netherlands_1987	99.3	99.3		97.1	97.1	97.1	97.1	96.8	96.4	99.0	99.0	98.6	99.0	98.3	97.7	96.8	96.9	96.8	96.7	96.7	96.9	96.9	96.7	96.7	96.7	96.7	96.7	96.7	96.8	96.8	96.8	96.7	96.6	97.1	97.1	97.1	96.3	96.0	94.9	95.4
4	PQ204794.1 China_2022	96.7	96.8	96.6		98.4	98.7	95.4	94.8	94.7	96.8	96.8	96.4	96.8	96.2	95.7	95.1	95.0	95.1	94.8	94.9	95.0	95.0	94.8	94.8	94.8	94.8	94.9	94.7	94.9	94.9	94.9	94.8	94.7	95.4	95.2	95.4	94.6	94.2	93.4	93.8
5	PQ204807.1 China_2023	96.7	96.7	96.5	98.1		98.6	95.5	94.8	94.6	96.9	96.8	96.4	96.8	96.2	95.9	95.2	95.1	95.2	95.0	95.1	95.1	95.1	95.0	95.0	95.0	95.0	95.1	94.9	95.1	95.1	95.1	95.0	94.8	95.5	95.4	95.5	94.6	94.3	93.4	93.8
6	PQ204810.1 China_2024	96.7	96.7	96.5	98.6	98.2		95.5	94.9	94.8	96.9	96.9	96.5	96.9	96.3	95.8	95.1	95.1	95.1	94.9	95.0	95.1	95.1	94.9	94.9	94.9	94.9	95.0	94.8	95.0	95.0	95.0	94.9	94.7	95.5	95.3	95.5	94.7	94.3	93.3	93.9
7	PV096984.1 China_2024	97.2	97.1	97.0	94.8	94.7	94.8		95.0	94.6	96.9	96.9	96.7	97.0	96.5	95.9	95.3	95.4	95.5	95.2	95.3	95.4	95.4	95.2	95.2	95.2	95.2	95.3	95.2	95.3	95.3	95.3	95.2	95.1	99.7	98.9	99.7	97.8	97.7	96.4	97.1
8	PP781502.1 South_Korea_2024	96.6	96.7	96.7	94.7	94.6	94.8	95.3		98.1	96.9	96.9	97.5	96.9	97.0	97.0	96.3	96.4	96.5	96.3	96.3	96.4	96.4	96.3	96.4	96.4	96.3	96.2	96.2	96.3	96.3	96.3	96.3	96.1	95.0	94.9	95.0	94.2	93.8	92.9	93.3
9	PP781503.1 South_Korea_2024	96.5	96.5	96.6	94.6	94.5	94.8	95.1	98.6		96.5	96.5	97.1	96.5	96.6	96.6	96.0	96.1	96.2	95.9	96.0	96.1	96.1	95.9	96.1	96.1	95.9	95.9	95.9	96.0	96.0	96.0	95.9	95.8	94.6	94.6	94.6	93.9	93.5	92.7	93.1
**10**	**PRCV isolate 89V367 Belgium_1989**	99.2	99.3	99.1	96.4	96.3	96.3	96.7	96.7	96.4		99.7	99.0	99.7	98.7	98.4	97.1	97.1	97.2	97.0	97.1	97.1	97.1	97.0	97.0	97.0	97.0	96.9	97.0	97.1	97.1	97.1	97.0	96.8	96.9	96.8	96.9	96.0	95.8	94.7	95.1
**11**	**PRCV isolate 89V431 Belgium_1989**	99.2	99.3	99.2	96.4	96.3	96.3	96.8	96.7	96.5	99.8		99.0	99.7	98.7	98.4	96.9	97.0	97.1	96.8	96.9	97.0	97.0	96.8	96.8	96.8	96.8	96.7	96.8	96.9	96.9	96.9	96.8	96.7	96.9	96.8	96.9	96.0	95.8	94.8	95.1
**12**	**PRCV isolate 90V170 Belgium_1990**	99.0	99.1	99.0	96.3	96.1	96.2	96.7	97.2	96.9	99.2	99.2		99.0	99.1	98.3	97.3	97.4	97.5	97.2	97.3	97.4	97.4	97.2	97.2	97.2	97.2	97.1	97.2	97.3	97.3	97.3	97.2	97.1	96.7	96.6	96.7	95.8	95.5	94.4	94.9
13	OK078898.1 Denmark_1990	99.2	99.3	99.2	96.4	96.3	96.3	96.8	96.7	96.5	99.7	99.7	99.2		98.7	98.6	96.9	97.0	97.1	96.8	96.9	97.0	97.0	96.8	96.8	96.8	96.8	96.7	96.8	96.9	96.9	96.9	96.8	96.7	97.0	96.9	97.0	96.1	95.9	94.7	95.2
14	OR689864.1 Belgium_1991	98.8	98.9	98.8	96.1	96.0	96.0	96.6	96.9	96.7	98.9	99.0	99.4	99.0		98.1	97.3	97.4	97.5	97.2	97.4	97.4	97.4	97.2	97.2	97.2	97.2	97.1	97.2	97.3	97.3	97.3	97.2	97.1	96.5	96.6	96.5	95.7	95.7	94.4	95.0
15	OR689863.1 Italy_2012	98.0	98.1	98.0	95.5	95.4	95.5	96.1	96.9	96.9	98.3	98.4	98.5	98.5	98.4		97.6	97.7	97.8	97.5	97.6	97.7	97.7	97.5	97.5	97.5	97.5	97.5	97.5	97.6	97.6	97.5	97.5	97.4	95.9	96.0	95.9	95.2	94.9	93.9	94.3
16	G/071/2020 Belgium_2020	96.6	96.7	96.7	94.4	94.4	94.4	95.1	95.9	95.8	96.7	96.7	97.2	96.7	97.2	95.0		99.9	99.1	99.8	98.9	99.9	99.9	99.8	99.7	99.7	99.8	99.7	99.6	99.8	99.8	99.8	99.6	99.6	95.3	95.3	95.3	94.3	94.2	93.5	93.8
17	G/721/2021 Belgium_2021	96.5	96.6	96.7	94.3	94.3	94.3	95.1	95.9	95.7	96.7	96.7	97.1	96.7	97.1	97.4	99.9		99.2	99.8	99.0	100	100	99.8	99.8	99.8	99.8	99.8	99.7	99.9	99.9	99.8	99.7	99.7	95.4	95.4	95.4	94.3	94.3	93.5	93.9
18	G/794/2021 The_Netherlands_2021	96.8	96.9	96.9	94.4	94.4	94.4	95.2	95.9	95.8	96.9	96.9	97.4	96.9	97.4	97.6	98.9	98.9		99.0	99.8	99.2	99.2	99.0	99.0	99.0	99.0	99.0	98.9	99.1	99.1	99.0	99.0	98.9	95.5	95.5	95.5	94.4	94.4	93.5	94.2
19	G/803/2021 The_Netherlands_2021	96.4	96.5	96.6	94.2	94.2	94.3	95.0	95.8	95.7	96.5	96.6	97.0	96.5	97.0	97.3	99.6	99.6	98.7		98.8	99.8	99.8	99.7	99.6	99.6	99.7	99.6	99.5	99.8	99.8	99.7	99.5	99.5	95.2	95.2	95.2	94.2	94.1	93.4	93.7
20	G/811/2021 The_Netherlands_2021	96.7	96.8	96.8	94.3	94.3	94.3	95.1	95.8	95.6	96.8	96.8	97.3	96.8	97.3	97.5	98.8	98.8	99.9	98.6		99.0	99.0	98.8	98.7	98.7	98.8	98.7	98.6	98.9	98.9	98.8	98.8	98.6	95.3	95.4	95.3	94.3	94.3	93.4	94.0
21	G/1146/2021 Belgium_2021	96.5	96.7	96.7	94.3	94.3	94.4	95.1	96.0	95.8	96.7	96.7	97.1	96.7	97.1	97.5	99.9	99.9	98.9	99.6	98.8		100	99.8	99.8	99.8	99.8	99.8	99.7	99.9	99.9	99.8	99.7	99.7	95.4	95.4	95.4	94.3	94.3	93.5	93.9
22	G/1538/2021 Germany_2021	96.5	96.6	96.6	94.3	94.3	94.3	95.0	95.9	95.6	96.6	96.6	97.1	96.6	97.1	97.4	99.8	99.7	98.8	99.5	98.7	99.8		99.8	99.8	99.8	99.8	99.8	99.7	99.9	99.9	99.8	99.7	99.7	95.4	95.4	95.4	94.3	94.3	93.5	93.9
23	G/2061/2021 Belgium_2021	96.5	96.6	96.7	94.2	94.3	94.3	95.1	95.8	95.7	96.6	96.7	97.1	96.6	97.1	97.4	99.9	99.8	98.8	99.6	98.7	99.8	99.7		99.6	99.6	99.7	99.6	99.5	99.8	99.8	99.7	99.5	99.5	95.2	95.2	95.2	94.2	94.1	93.4	93.7
24	G/2269/2022 Belgium_2022	96.5	96.6	96.6	94.3	94.3	94.3	95.0	95.9	95.7	96.6	96.6	97.1	96.6	97.0	97.3	99.7	99.7	98.7	99.4	98.6	99.7	99.5	99.6		100	99.6	99.5	99.6	99.7	99.7	99.6	99.4	99.4	95.2	95.2	95.2	94.2	94.1	93.4	93.7
25	G/2271/2022 Belgium_2022	96.5	96.6	96.6	94.3	94.3	94.3	95.0	95.9	95.7	96.6	96.6	97.1	96.6	97.0	97.3	99.7	99.7	98.7	99.4	98.6	99.7	99.5	99.6	100		99.6	99.5	99.6	99.7	99.7	99.6	99.4	99.4	95.2	95.2	95.2	94.2	94.1	93.4	93.7
26	G/3063/2022 Belgium_2022	96.5	96.6	96.6	94.2	94.2	94.2	95.0	95.8	95.7	96.6	96.6	97.1	96.6	97.1	97.4	99.8	99.8	98.8	99.5	98.7	99.8	99.7	99.8	99.6	99.6		99.6	99.5	99.8	99.8	99.7	99.5	99.5	95.2	95.2	95.2	94.2	94.3	93.5	93.9
27	G/2196/2023 Belgium_2023	96.4	96.5	96.5	94.3	94.3	94.3	95.0	95.8	95.6	96.5	96.5	97.0	96.5	97.0	97.3	99.8	99.7	98.7	99.4	98.6	99.7	99.6	99.7	99.6	99.6	99.7		99.4	99.7	99.7	99.6	99.4	99.4	95.3	95.3	95.3	94.3	94.2	93.4	93.8
28	Gent/85-3/2023 Belgium_2023	96.4	96.5	96.5	94.2	94.2	94.2	95.0	95.8	95.6	96.6	96.6	97.0	96.5	96.9	97.3	99.7	99.6	98.6	99.3	98.5	99.7	99.5	99.6	99.5	99.5	99.5	99.5		99.6	99.6	99.5	99.4	99.4	95.2	95.2	95.2	94.2	94.1	93.4	93.6
29	G/86-1/2023 Belgium_2023	96.5	96.6	96.7	94.2	94.3	94.3	95.0	95.9	95.7	96.6	96.7	97.1	96.6	97.1	97.4	99.8	99.7	98.8	99.4	98.7	99.7	99.6	99.7	99.6	99.6	99.7	99.7	99.5		99.8	99.8	99.6	99.6	95.3	95.3	95.3	94.3	94.2	93.5	93.8
30	G/87-4/2023 Belgium_2023	96.3	96.4	96.5	94.1	94.2	94.2	94.9	95.7	95.6	96.5	96.5	96.9	96.5	96.9	97.3	99.7	99.7	98.7	99.4	98.6	99.7	99.5	99.6	99.5	99.5	99.6	99.5	99.7	99.5		99.8	99.6	99.6	95.3	95.3	95.3	94.3	94.2	93.5	93.8
31	G/90-6/2023 Belgium_2023	96.4	96.5	96.5	94.2	94.2	94.2	95.0	95.9	95.7	96.5	96.5	97.0	96.5	97.0	97.3	99.7	99.6	98.7	99.4	98.6	99.7	99.5	99.6	99.5	99.5	99.6	99.6	99.4	99.7	99.4		99.5	99.5	95.3	95.3	95.3	94.3	94.2	93.5	93.8
32	G/91-1/2023 Belgium_2023	96.4	96.5	96.6	94.3	94.2	94.2	95.0	95.8	95.6	96.5	96.6	97.0	96.5	97.0	97.3	99.7	99.7	98.7	99.4	98.6	99.7	99.5	99.6	99.5	99.5	99.6	99.5	99.4	99.5	99.5	99.4		99.4	95.2	95.2	95.2	94.2	94.1	93.4	93.7
33	G/95-1/2023 Belgium_2023	96.3	96.4	96.5	94.1	94.1	94.1	94.9	95.7	95.5	96.5	96.5	96.9	96.5	96.9	97.2	99.7	99.7	98.7	99.4	98.6	99.7	99.5	99.6	99.5	99.5	99.6	99.5	99.4	99.5	99.5	99.4	99.5		95.1	95.1	95.1	94.0	94.0	93.3	93.6
34	OR209251.1 USA_1989	97.0	97.0	96.8	94.8	94.7	94.8	99.5	94.8	94.7	96.6	96.6	96.5	96.6	96.4	95.8	94.9	94.8	94.9	94.7	94.8	94.9	94.7	94.8	94.7	94.7	94.7	94.7	94.7	94.8	94.6	94.8	94.7	94.6		99.2	100	98.2	97.7	96.7	97.1
35	OM830321.1 USA_1990	96.9	96.9	96.7	94.8	94.6	94.8	98.8	94.6	94.6	96.5	96.5	96.4	96.5	96.4	95.7	94.8	94.8	94.8	94.6	94.7	94.8	94.7	94.7	94.6	94.6	94.7	94.6	94.6	94.7	94.5	94.6	94.6	94.6	99.3		99.2	98.0	98.0	97.0	97.5
36	OR209252.1 USA_1991	97.0	97.0	96.8	94.8	94.7	94.8	99.5	94.8	94.7	96.6	96.6	96.5	96.6	96.4	95.8	94.9	94.8	94.9	94.7	94.8	94.9	94.7	94.8	94.7	94.7	94.7	94.7	94.7	94.8	94.6	94.8	94.7	94.6	100	99.3		98.2	97.7	96.7	97.1
37	OR209253.1 USA_1992	96.7	96.7	96.5	94.5	94.3	94.4	97.9	94.4	94.3	96.3	96.4	96.3	96.4	96.3	95.5	94.4	94.4	94.4	94.2	94.3	94.3	94.3	94.3	94.3	94.3	94.3	94.2	94.2	94.3	94.1	94.3	94.2	94.2	98.4	98.2	98.4		96.7	95.9	96.1
38	KR270796.1 USA_2014	94.8	94.7	94.6	93.1	93.1	93.0	96.5	92.7	92.7	94.4	94.4	94.3	94.5	94.5	93.7	93.1	93.1	93.2	93.0	93.1	93.0	92.9	93.0	93.0	93.0	93.0	92.9	92.9	93.0	92.9	92.9	92.9	92.8	96.8	97.0	96.8	96.0		96.0	98.4
39	KY406735.1 USA_2016	94.8	94.7	94.7	92.7	92.8	92.8	96.5	92.7	92.7	94.4	94.5	94.3	94.5	94.3	93.6	93.0	93.0	93.0	92.8	92.9	93.0	92.9	93.0	92.9	92.9	92.9	92.9	92.8	92.9	92.8	92.9	92.8	92.8	97.0	97.3	97.0	96.1	96.3		95.7
40	OR209254.1 USA_2020	94.4	94.4	94.3	93.0	93.1	92.8	96.1	92.6	92.5	94.1	94.1	94.0	94.2	94.1	93.3	92.6	92.6	92.8	92.5	92.7	92.6	92.4	92.6	92.5	92.5	92.5	92.4	92.4	92.5	92.4	92.4	92.4	92.3	96.3	96.6	96.3	95.5	98.0	95.9	

Figure S1E: A heatmap of the nucleotide (lower left segment) and amino acid (upper right segment) sequence identities of the PRCV S protein of isolates circulating in Belgium, Germany and the Netherlands from 2020 to 2023 compared to other sequences retrieved from GenBank. The isolates in bold are from archived Belgian isolates. The colour scale indicates the percent nucleotide (lower left) and aa (upper right) identities. Classical strains including PRCV isolate TLM83, are shaded in orange, Asian strains in turquoise, European strains in blue and strains from North America in pink. The strains obtained in this study are highlighted in red text.
